# Effect of age at vaccination on the measles vaccine effectiveness and immunogenicity: systematic review and meta-analysis

**DOI:** 10.1186/s12879-020-4870-x

**Published:** 2020-03-29

**Authors:** Sara Carazo, Marie-Noëlle Billard, Amélie Boutin, Gaston De Serres

**Affiliations:** 1 Department of Social and Preventive Medicine, Laval University, 1050, Avenue de la Médecine, Quebec, QC G1V 0A6 Canada; 2 CHU de Québec – Université Laval Research Center, 2400, Avenue d’Estimauville, Quebec, QC G1E 7G9 Canada; 3 Institut National de Santé Publique du Québec, 2400, Avenue d’Estimauville, Quebec, QC G1E 7G9 Canada

**Keywords:** Measles vaccine, Age, Immunogenicity, Effectiveness

## Abstract

**Background:**

The objectives of this review were to evaluate the effect of age at administration of the first dose of a measles-containing vaccine (MCV1) on protection against measles and on antibody response after one- and two-dose measles vaccinations.

**Methods:**

We conducted a systematic review of the PubMed/MEDLINE, Embase, Web of Science and Cochrane databases (1964–2017) to identify observational studies estimating vaccine effectiveness and/or measles attack rates by age at first vaccination as well as experimental studies comparing seroconversion by age at first vaccination. Random effect models were used to pool measles risk ratios (RR), measles odds ratios (OR) and seroconversion RR of MCV1 administered at < 9, 9–11 or ≥ 15 months compared with 12 or 12–14 months of age.

**Results:**

We included 41 and 67 studies in the measles protection and immunogenicity analyses. Older age at MCV1, from 6 to ≥15 months, improved antibody response and measles protection among one-dose recipients. Pooled measles RR ranged from 3.56 (95%CI: 1.28, 9.88) for MCV1 at < 9 months to 0.48 (95%CI: 0.36, 0.63) for MCV1 at ≥15 months, both compared to 12–14 months. Pooled seroconversion RR ranged from 0.93 (95%CI: 0.90, 0.96) for MCV1 at 9–11 months to 1.03 (95%CI: 1.00, 1.06) for MCV1 at ≥15 months, both compared to 12 months. After a second dose, serological studies reported high seropositivity regardless of age at administration of MCV1 while epidemiological data based on few studies suggested lower protection with earlier age at MCV1.

**Conclusions:**

Earlier age at MCV1 decreases measles protection and immunogenicity after one dose and might still have an impact on vaccine failures after two doses of measles vaccine. While two-dose vaccination coverage is most critical to interrupt measles transmission, older age at first vaccination may be necessary to keep the high level of population immunity needed to maintain it.

## Background

The introduction of measles vaccination in the 1960s helped to control this highly contagious disease [1]. Elimination was achieved in the Americas in 2002 and the World Health Organization (WHO) has set the goal of measles elimination [2].

In one-dose programs, vaccine effectiveness (VE) was influenced by age at vaccination [3–5]. The interference of maternal antibodies and the immaturity of the child’s immune system were the alleged mechanisms that resulted in a weaker antibody response and poorer protection in younger infants [6–9]. A second dose of MCV (MCV2) was added to compensate for the primary failures observed after the first vaccination, and two-dose schedules have been a key strategy for measles elimination [2]. However, epidemic investigations [10, 11] and serological studies [12] have suggested that the effect of age at MCV1 could persist after two doses, with increased vulnerability among children first vaccinated before 15 months.

The recommended age at first dose is a compromise, balancing the advantage of older immunization with the risk of measles in infants [13]. Depending upon the country, the first dose of measles-containing vaccine (MCV1) is currently administered from 9 to 18 months of age, with vaccination recommended as early as 6 months in specific situations [14–16].

In their systematic review, Uzicanin and Zimmerman [17] reported a VE of 84 to 93% after one and of 94% after two doses of vaccine. They presented VE summary point estimates for one dose administered at 9–11 (77%) or ≥ 12 months (92%). A Cochrane review studied the effectiveness and safety of the measles-mumps-rubella vaccine but without accounting for age at vaccination [18]. Finally, a recent review examined immunogenicity and effectiveness of vaccination at < 9 months [19]. None of them have systematically reviewed the impact of a change in age at MCV1 on the vaccine response. In order to control measles or to maintain measles elimination, public health stakeholders have to decide on best vaccination schedules based on their country’s epidemiology, health system characteristics and best evidence on the effect of age at vaccination.

We aimed to evaluate the effect of age at administration of MCV1 on protection against measles and antibody response after one- and two-dose measles vaccinations through a systematic review of observational studies estimating VE and/or measles attack rates (AR) by age at first vaccination as well as experimental studies comparing seroconversion risk by age at first vaccination.

## Methods

We conducted a systematic review of the literature following the Cochrane Handbook for Systematic Reviews of Interventions [20] methodological recommendations, and we reported our results according to the Preferred Reporting Items for Systematic Reviews and Meta-Analyses (PRISMA) statement [21].

The study protocol is available in Additional file 1.

### Eligibility criteria

Eligibility required evaluation of vaccination with one or two doses of further attenuated live MCV. In each study, the first dose had to be administered at different ages, but all before the age of two years.

Cohort and case-control studies that reported VE and measles AR by age at MCV1 were eligible for the review of measles protection. Studies or participants vaccinated during an outbreak were excluded.

Randomized controlled trials (RCT) and quasi-experimental studies were included in the review of immunogenicity if seroconversion after MCV1 and/or seropositivity after MCV2 were reported by age at first vaccination. As both age and antibody detection were objective measures, quasi-experimental designs were thought to give valuable data on the immunogenicity response to measles vaccine. Studies of killed and high titer vaccines were excluded, as well as those examining aerosol or intradermal administration or targeting populations with special characteristics such as immunosuppressed or malnourished children [22]. When different vaccine strains were administered in one study, results were extracted according to the strains to compare children receiving the same strain at different ages.

### Search strategy

Studies were identified by a systematic search of the PubMed/MEDLINE, Embase, Web of Science and Cochrane databases from 1964, when the first measles vaccine was licensed, to May 2017. Reference lists of selected articles and key published reviews [17–19, 23] were also hand-searched.

The following search terms were included: Measles Vaccine, Measles-Mumps-Rubella Vaccine, Measles/prevention and control, Vaccination, Measles Mumps Rubella Varicella vaccine, MMR, MMRV, Vaccine effectiveness, Efficacy, Epidemic, Outbreak, Treatment failure, Vaccine failure, Antibody, Serologic Tests, Seroconversion, Immunogenicity, Age, Age at vaccination, Age at immunization and Age factor. The search strategy, validated by a professional librarian, was adapted to each database. Search results were limited to human studies. Studies published in English, French, Spanish or Portuguese were included (Additional file 1).

### Study selection

After eliminating duplicates using EndNote version X7.1 (New York City: Thomson Reuters, 2011) and manual completion, two reviewers (SCP and MNB) independently selected the studies based on the criteria described here. Any disagreements were resolved by consensus or consulting a third party (GDS). The main reason for exclusion was recorded during the full-text examination.

### Data collection process

Data extraction forms were developed and tested for each sub-analysis (measles protection or immunogenicity).

A single author (SCP or MNB) abstracted data from the studies, which were checked by a second reviewer (SCP or MNB). Authors of original articles were contacted in the event of missing or inaccurate information. When VE or seroconversion risk was not reported but there were enough data to estimate it by age at vaccination, the calculation was done by the reviewers.

### Data items

We extracted information on the study’s characteristics, population, intervention (vaccine strain, age at vaccination, number of doses, interval between doses and vaccine status ascertainment) and outcome (case definition, seroconversion definition, antibody assay methods, attack rates and seroconversion risk). VE was calculated by comparing measles AR among vaccinated and non-vaccinated or comparing the vaccination status of cases and non-cases during measles epidemics [24, 25]. Measles cases were defined by clinical, epidemiological and/or serological criteria. Attenuated or non-classical measles cases were included only if confirmed by laboratory [13]. Seroconversion was defined, according to the study, as the presence of measles antibodies in individuals with previously undetectable titers, a fourfold increase in their concentration or both. Seropositivity was defined as an antibody concentration higher than the protective threshold [26]. Antibody assay methods included enzyme immunoassays (ELISA), hemagglutination assays (HAI), plaque reduction neutralization tests (PRN) and complement fixation tests (CF) [27, 28].

### Risk of bias of individual studies

Two reviewers (SCP and MNB) independently evaluated the risk of bias for each outcome. For the assessment of observational studies, a scale was adapted from the NICE public health guidance [29] to evaluate the study’s representativeness, selection process, comparability of the groups, vaccination status ascertainment, and outcome definition and completeness [25]. The Cochrane collaboration tool [20] was adapted to evaluate the risk of bias in experimental studies (Additional file 1).

### Summary measures and synthesis of results

To summarize measles protection studies, we reported estimates of VE conferred by MCV1 and MCV2 for each age group, and also presented measles risk ratios (RR) or odds ratios (OR) using the age group containing children aged 12 months as the referent group. Immunogenicity was presented as seroconversion risk after MCV1 and seropositivity risk after MCV2 for each age category, as reported in each study. Age groups with less than 10 participants were combined if not including the 12 months category.

Meta-analyses of cohort or case-control studies were conducted to pool RR or OR of measles by age at first vaccination. Studies were included if reporting measles AR among 12 or 12 to 14-month-old children, the reference category, and one of the following comparison groups: < 9 months, 9 to 11 months, < 12 months or ≥ 15 months. These age categories were chosen based on current vaccination policies [14–16].

For the immunological studies the reference age category was 12 months, with the same comparison age groups. RR of seroconversion by age at first vaccination were pooled separately according to the post-MCV1 seroconversion definition (fourfold increase in antibody titer, seropositivity among previously seronegative or both). Overall measures of association were reported only if subgroup differences were not significant. All meta-analyses were performed using random effect models in Review Manager version 5.3 (Copenhagen: The Cochrane Collaboration, 2014). Heterogeneity between studies was evaluated using I^2^ and considered significant if greater than 50% [30]. Heterogeneity was explored according to the following a priori identified factors: measles case definitions, WHO regions and year of epidemic for the measles protection analysis; and vaccine strain, antibody assay method and year of the study for the immunogenicity analysis.

### Risk of bias across studies

Publication bias was evaluated for each outcome by visual examination of funnel plots.

### Additional analyses

Pre-specified sensitivity analyses of studies with a low risk of bias and of RCT were conducted when appropriate. Finally, a pooled analysis comparing seroconversion after MCV1 administered at 6 versus 9 months was performed a posteriori, considering that there is a recommendation for vaccination at 6 months in the context of epidemics in countries with high measles mortality [14].

## Results

### Study selection

**Fig. 1:**
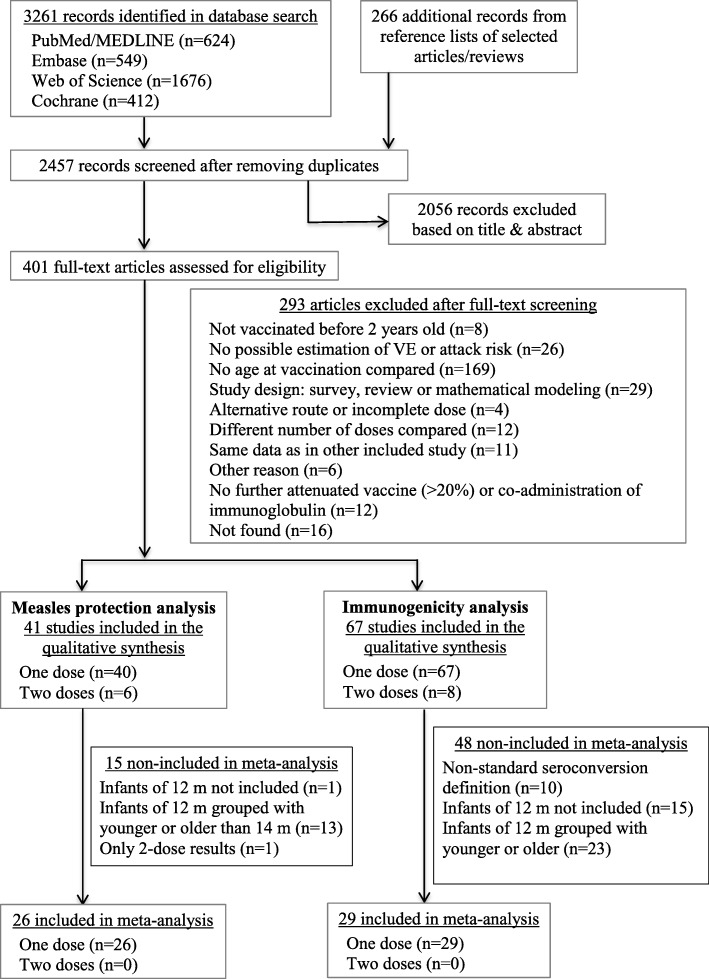
Study selection flow diagram

After removing duplicates, we screened 2723 references and 108 studies were included in the review (Fig. 1). Selection agreement was > 90%.

### Study characteristics

Of the 41 studies included in the measles protection analysis, there were 29 retrospective cohort and 12 case-control studies (Table 1 and Additional file 2) [3–5, 10, 11, 31–67]. Most were conducted in the Americas (*n* = 21; 51%), Africa (*n* = 5; 12%) and Western Pacific (*n* = 5; 12%) regions and 59% reported school epidemics. Vaccination status was verified by written record in 36 studies (88%) but only 12 studies (29%) included solely laboratory confirmed cases or epidemiologically linked cases. Although 29 studies were conducted in large epidemics (≥100 cases), only 11 (27%) reported > 100 cases with data on vaccination status and age and 2 (5%) presented less than 10 cases.

**Table 1:** Vaccine effectiveness and measles risk ratio by age at first vaccination

A. ONE-DOSE ANALYSIS A-1. VACCINE EFFECTIVENESS [95% Confidence Intervals]									
Author, year of epidemic (ref)	Cases^a^		Age at first vaccination						
			< 9	9	10–11	12	13–14	15	> 15
McIntyre, 1977 [31]	65					100% [−]	75.2% [47, 96]	93.3% [87, 97]	
Aaby, 1980 [32]	49		54.1% [5, 78]	48.6% [0, 76]		69.8% [49, 82]			
Hull, 1981 [33]	61		41.9% [0, 76]	85.5% [56, 95]		85.9% [45, 96]		90.2% [78, 96]	
Anonymus, 1983 [34]	264		43.0% [NC]	83.0% [NC]					
Nkowane, 1984 [35]	18		33.3% [0, 83]			98.0% [82, 100]		93.2% [78, 98]	
McCombie, 1985 [36]	83		−87.9% [− 438, 34]			33.0% [0, 73]		51.0% [0, 79]	
Davis, 1985 [37]	25		100% [−]			97.5% [91, 99]		96.8% [83, 99]	
Roberston, 1986 [38]	40		70.2% [22, 80]			84.0% [63, 93]		95.1% [88, 98]	
George, 1986 [39]	67		50.3% [22, 68]	59.0% [36, 74]					
Sharma 1987 [40]	132			49.7% [22, 68]	52.7% [23, 71]		71.0% [0–96]	73.4% [0, 96]	
Hersh, 1987 [41]	46		80.8% [39, 94]			80.5% [51, 92]		93.7% [84, 97]	
Lee, 1988 [42]	52		79.5% [0–97]				75.4% [3, 94]		78.9 [61, 88]
Chawla, 1989 [3]	176			77.3% [53, 89]		87.7% [67, 95]		100% [−]	
Rivest, 1989 [4]	138				87.2% [0, 99]	91.5%[0, 100]	96.5% [37, 100]	97.8% [60, 100]	
De la Puente,1990 [43]	50					58.5% [13, 80]		70.5% [59, 79]	
Malfait, 1990 [44]	94			25.9% [8, 41]		94.4% [93, 96]			
Murray, 1990 [5]	74		40.1% [0, 81]	75.5% [47, 89]		86.9% [66, 95]		92.0% [75, 97]	
Coetzee, 1992 [45]	17		74.7% [13, 93]				81.4% [51–93]		
McDonnell,1993 [46]	40		96.6% [66, 100]			95.7% [84, 99]		95.0% [84, 98]	
Lee, 1994 [47]	6		30.0% [0, 87]			72.0% [0, 95]		100% [−]	
Kaninda, 1995 [48]	1295		86.9% [81, 91]	94.2% [93, 95]					
Kotb, 1995 [49]	230			25.6%[0, 53]	72.3% [55, 83]				
Hennessey, 1996 [50]	167		91.5% [67, 98]	87.9% [83–92]		91.0% [87, 94]			90.0 [84, 94]
John, 1999 [51]	67		24.0% [0, 55]	62.2% [0, 87]					
A-2. MEASLES RISK RATIO / ODDS RATIO [95% Confidence Intervals]									
Author, year of epidemic (ref)	Cases^a^	RR/OR^b^	Age at first vaccination						
			< 9	9	10–11	12	13–14	15	> 15
Shelton, 1976 [52]	21	OR	1.00 [0.2, 4.7]			1.00 (ref)		0.05 [0.01, 0.3]	
Judelsohn, 1978 [53]	58	RR	1.92 [0.7, 5.3]		0.49 [0.2, 1.1]	1.00 (ref)	0.34 [0.1, 0.8]	0.48 [0.3, 0.9]	
Faust, 1978 [54]	63	RR	0.92 [0.5, 1.6]			1.00 (ref)	0.26 [0.1, 0.6]	0.44 [0.1, 1.4]	0.25 [0.1, 0.7]
Lopes, ≈1979 [55]	14	RR	1.62 [0.7, 3.6]			1.00 (ref)			
Aaby, 1980 [32]	49	RR	1.52 [0.6, 3.6]	1.70 [0.7, 4.2]		1.00 (ref)			
Hull, 1981 [33]	61	RR	4.13 [0.8, 20.2]	1.03 [0.2, 5.8]		1.00 (ref)		0.69 [0.2, 3.3]	
Wassilak, 1981 [56]	18	OR	1.00 [0.1, 8.4]			1.00 (ref)	0.20 [0.02, 2.0]	0.11 [0.03, 0.4]	
Hull, 1984 [57]	21	OR	NC			1.00 (ref)		0.21 [0.1, 0.9]	
Nkowane, 1984 [35]	18	RR	32.92 [3.8, 283.5]			1.00 (ref)		3.31 [0.4, 25.7]	
McCombie, 1985 [36]	83	RR	2.80 [1.2, 6.4]			1.00 (ref)		0.73 [0.4, 1.2]	
Davis, 1985 [37]	25	RR	0			1.00 (ref)		1.30 [0.4, 4.4]	
Chen, 1985 [58]	16	OR	1.20 [0.2, 7.3]			1.00 (ref)		0.14 [0.03, 0.6]	
Mast, 1986 [59]	170	OR	3.09 [0.9, 1.08]			1.00 (ref)		0.15 [0.1, 0.3]	
Roberston, 1986 [38]	40	RR	1.86 [0.9, 3.9]			1.00 (ref)		0.31 [0.2, 0.7]	
Sharma 1987 [40]	132	RR		1.06 [0.6, 2.0]	1.00 (ref)		0.61[0.1, 4.2]	0.56 [0.1, 3.9]	
Hutchins, 1987 [60]	30	RR				1.00 (ref)	0.49 [0.1, 1.8]	0.60 [0.3, 1.3]	
Hersh, 1987 [41]	46	RR	0.99 [0.4, 2.6]			1.00 (ref)		0.32 [0.2, 0.6]	
Agocs, 1988 [61]	79	RR	1.00 (ref)				0.92 [0.6, 1.4]		
Lee, 1988 [42]	52	RR	1.00 (ref)				1.20 [0.1, 12.4]		1.03 [0.1, 7.5]
Paunio, 1988 [10]	153^c^	OR	1.00 (ref)				0.37 [0.1, 1.0]		
Chawla, 1989 [3]	176	RR		1.84 [0.6, 6.1]		1.00 (ref)		0.00	
Ng, 1989 [62]	16	RR	1.70 [0.4, 7.2]			1.00 (ref)		0.41 [0.1, 3.1]	
De Serres, 1989 [63]	525	OR	0.98 [0.7, 1.4]			1.00 (ref)	0.50 [0.4, 0.7]	0.29 [0.2, 0.4]	
Rivest, 1989 [4]	138	OR			1.56 [0.5, 4.5]	1.00 (ref)	0.40 [0.2, 0.8]	0.26 [0.2, 0.5]	
De la Puente, 1990 [43]	50	RR				1.00 (ref)		0.71 [0.3, 1.7]	
Malfait, 1990 [44]	94	RR		13.23 [6.4, 26.5]		1.00 (ref)			
De Serres, 1990 [64]	5514	OR			1.40 [1.2, 1.6]	1.00 (ref)	0.59 [0.5, 0.6]	0.38 [0.3, 0.4]	0.38 [0.3, 0.4]
Murray, 1990 [5]	74	RR	4.58 [1.1, 20.0]	1.88 [0.6, 6.2]		1.00 (ref)		0.62 [0.1, 2.6]	
Coetzee, 1992 [45]	17	RR	1.00 (ref)				0.73 [0.2, 2.8]		
McDonnell, 1993 [46]	40	OR	0.79 [0.1, 7.6]			1.00 (ref)		1.16 [0.4, 3.4]	
Patel, 1994 [65]	7	RR	0.00	3.6[0.4,29.3]	1.72[0.1, 25.9]	1.00 (ref)			
Lee, 1994 [47]	6	RR	2.50 [0.4, 15.4]			1.00 (ref)		0.00	
Sutcliffe, 1995 [66]	82	RR				1.00 (ref)		0.28 [0.2, 0.4]	
Kaninda, 1995 [48]	1295	RR	2.27 [1.5, 3.3]	1.00 (ref)					
Kotb, 1995 [49]	230	OR		2.69[1.9,3.9]	1.00 (ref)				
Hennessey, 1996 [50]	167	RR	0.91 [0.2, 3.7]	1.30 [0.8, 2.1]		1.00 (ref)			1.08 [0.6, 1.9]
John, 1999 [51]	67	RR	2.01 [0.8, 5.4]	1.00 (ref)					
B. TWO-DOSE ANALYSIS B-1. VACCINE EFFECTIVENESS [95% Confidence Intervals]									
Author, year of epidemic (ref)	Cases^a^		Age at first vaccination						
			< 9	9	10–11	12	13–14	15	> 15
McCombie, 1985 [36]	22		63.3% [0, 88]			55.1% [0, 89]		37.6% [0, 79]	
Roberston, 1986 [38]	4		72.2% [0, 96]			100% faf[−]			
Hersh, 1987 [41]	4		88.9% [12, 99]			100% [−]			
De Serres, 2011 [67]^d^	52					93.0% [90, 95]	94.7% [90, 97]	97.5% [94, 99]	
B-2. MEASLES RISK RATIO / ODDS RATIO [95% Confidence Intervals]									
Author, year of epidemic (ref)	Cases^a^	RR/OR^b^	Age at first vaccination						
			< 9	9	10–11	12	13–14	15	> 15
McCombie, 1985 [36]	22	RR	0.82 [0.2, 3.2]			1.00 (ref)		1.39 [0.4, 5.3]	
Paunio 1988 [10]	153^c^	OR	1.00 (ref)				0.29 [0.04, 2.0]		
De Serres, 1990 [64]	28	OR			3.48 [1.4, 8.4]	1.00 (ref)		1.27 [0.4, 4.0]	
Defay, 2011 [11]	99	OR				1.00 (ref)	1.04 [0.7, 1.7]	0.00	0.17[0.04,0.7]

Abbreviations: *NC* not calculable, *RR* relative risk, *OR* odds ratio

^a^ Number of cases included in the calculation of the age effect

^b^ Risk ratios comparing attack rates by age in months of administration of the first dose of measles vaccine (reference age category is the one containing 12 months, specified according to the paper). Odds ratios have been calculated for case-control studies

^c^ Number of cases in the study, not specified the number in the one and two dose analysis

^d^ Effectiveness assessed in a school outbreak during the epidemic reported in Defay, 2011

Of the 67 trials included in the immunogenicity analysis, 8 were RCT, 25 non-RCT and 34 before-after studies (Additional file 2) [6–8, 68–130]. They were conducted in Africa (*n* = 22; 33%), the Americas (*n* = 21; 31%), Western Pacific (*n* = 10; 15%) and other regions (*n* = 14; 21%). Participants were mainly vaccinated with Schwarz (*n* = 34; 42%) and Moraten strains (n = 21; 30%) while antibodies were measured using HAI (*n* = 40; 60%), ELISA (*n* = 12; 18%), PRN (*n* = 9; 13%) or other assays (*n* = 6; 9%). Authors defined seroconversion as a fourfold increase in titers (*n* = 10; 15%), seropositivity among seronegative pre-vaccination (*n* = 36; 54%), both (*n* = 11; 16%) or other (*n* = 10; 15%). Sample size varied from 21 to 1633 participants and 72% include > 100 vaccinated children.

### Risk of bias within studies

All observational studies were considered to have good representativeness. Only 10 (24%) had an overall low risk of bias while 14 (34%) and 17 (41%) presented a moderate or high risk, respectively (Additional file 3). The main biases identified were: selection bias due to potential measles history among non-cases [39, 45, 54]; misclassification due to parents’ definition of measles case [3, 5, 32, 33, 40, 48, 49, 55] and differential verification of vaccination status for cases and non-cases [54]; and confounding bias due to lack of adjustment for time since vaccination [34, 38, 47, 51, 64]. Among studies included in the meta-analysis, 7 (27%) were at low, 12 (46%) at moderate, and 7 (27%) at high risk of bias.

Half of the 8 RCT reporting immunogenicity had a low risk of bias overall [8, 68–70] while 4 presented a moderate risk of bias [71–74]. In quasi-experimental studies, 19 (32%) were considered at low, 26 (44%) at moderate, and 14 (24%) at high risk of bias overall (Additional file 4). Substantial risk of bias was mainly due to potential differential exposure to measles during the study [71, 75–78] and to information bias caused by the use of dried blood spots to measure antibodies [77, 79–91]. Risk of bias of studies included in the meta-analysis was assessed as low in 3 RCT and 6 Non-RCT, moderate in 1 RCT and 8 Non-RCT, and high in 1 Non-RCT.

### Measles protection by age at first vaccination

Of the 41 observational studies included, 24 allowed VE estimates by age at vaccination among one-dose recipients. All 7 studies comparing vaccination at 9–11 months (VE range: 25.9–87.9%) versus ≥12 months (VE range: 69.8–94.4%) found better protection for the latter category (682 cases). Protection was lower in 11 of 13 studies for those vaccinated before (VE range: 33.3–88.2%) rather than after 12 months (VE range: 69.8–96.1%) (869 cases) and similar in 2 studies (65 cases). VE was lower when the vaccine was administered at 12–14 months (VE range: 58.5–91.5%) compared to ≥15 months (VE range: 70.5–100%) in 8 of 12 studies (651 cases); it was similar in 2 studies and lower at ≥15 months in 2 studies. VE estimations from a school epidemic ranged from − 88% (MCV1 at < 12 months) to 51% (MCV1 at ≥15 months) and were considered biased due to misclassification and lower exposure of unvaccinated children [36] (Table 1A).

**Fig. 2:**
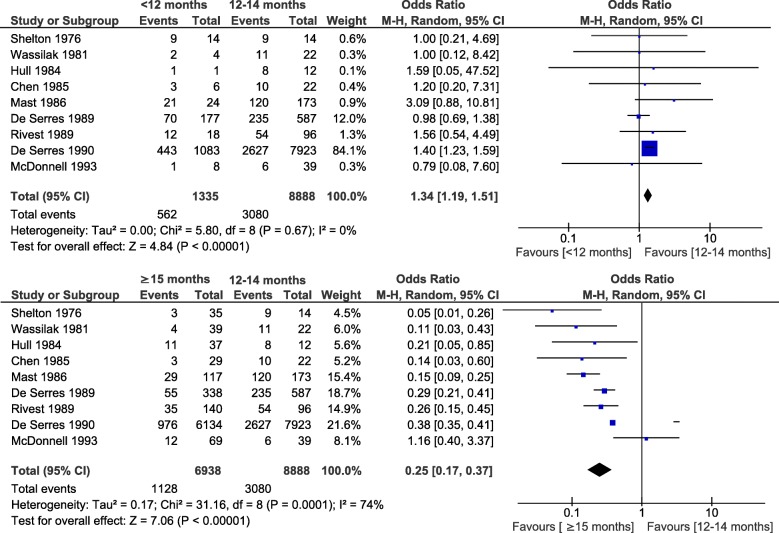
Age at first dose of measles-containing vaccine and risk of measles (cohort studies)

**Fig. 3:**
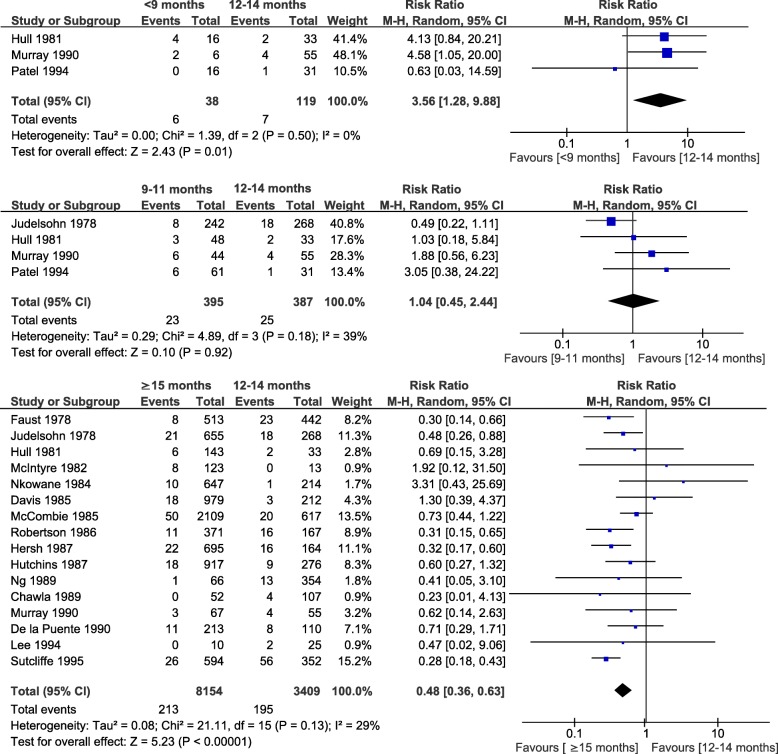
Age at first dose of measles-containing vaccine and risk of measles (case-control studies)

Measles RR/OR by age at vaccination could be estimated in 38 studies, 26 of which reported the defined age categories and were included in the meta-analysis. In the pooled analyses of cohort studies, vaccination before 9 months (RR = 3.56, 95%CI: 1.281, 9.88; I^2^ = 0%; 3 studies), at 9 to 11 months (RR = 1.04, 95%CI: 0.45, 2.44; I^2^ = 39%; 4 studies) or at < 12 months (RR = 1.62, 95%CI: 1.08, 2.43; I^2^ = 41%; 13 studies) were associated with a higher risk of measles compared to vaccination at 12–14 months. Conversely, vaccination at ≥15 months was associated with a lower risk than vaccination at 12–14 months (RR = 0.48, 95%CI: 0.36, 0.63; I^2^ = 29%; 16 studies) (Fig. 2 and Additional file 5). Similarly, meta-analysis of case-control studies showed a higher risk of measles with early MCV1: < 12 months versus 12–14 months (OR = 1.34, 95%CI: 1.19, 1.51; I^2^ = 0%; 9 studies); and a lower risk for MCV1 at ≥15 months (OR = 0.25, 95%CI: 0.17, 0.37; I^2^ = 74%; 9 studies) (Fig. 3). This last meta-analysis showed significant heterogeneity that was not explained by the predefined factors. The level of evidence of this analysis was considered low to moderate since it was based on observational studies in which a moderate risk of bias persisted but a “dose-response” trend was observed as older age at vaccination (from < 9 months to ≥15 months) was associated with lower measles risk [131].

Sensitivity analyses with cohort studies at low risk of bias included only 2 of the 18 studies, and only one for each comparison. Case-control studies at low risk of bias (5 out of 9) found a non-significant association comparing vaccination at < 12 versus 12 months (OR = 1.03, 95%CI: 0.75, 1.41) and a lower measles risk for vaccination at ≥15 versus 12 months (OR = 0.31, 95%CI: 0.19, 0.50), similar to that of the main analysis (Additional file 5).

Among two-dose recipients, two studies found lower VE if MCV1 was received at < 12 months versus at ≥12 months (72 and 89% versus 100%) but based on only one twice-vaccinated case in each study [38, 41]. The largest effectiveness study among two dose recipients (52 cases) reported VE of 93 and 98% for those first vaccinated at 12 and ≥ 15 months [67]. Results of 3 case-control studies showed: a lower risk of measles if MCV1 was administered at ≥15 months versus 12 months (OR = 0.12, 95%CI: 0.03, 0.51) [11]; a non-significant lower risk comparing ≥14 months versus < 14 months (OR = 0.29, 95%CI: 0.04, 2.00) [10]; and a higher risk for those first vaccinated at 10–11 months (OR = 3.48, 95%CI: 1.4, 8.4) and at ≥15 months (OR = 1.27, 95%CI: 0.41, 3.98) compared to 12–14 months [64] (Table 1B).

### Immunogenicity by age at first vaccination

Of the 67 studies reporting on one-dose immunogenicity, 19 fulfilled the criteria to be included in the meta-analysis (Fig. 1).

**Fig. 4:**
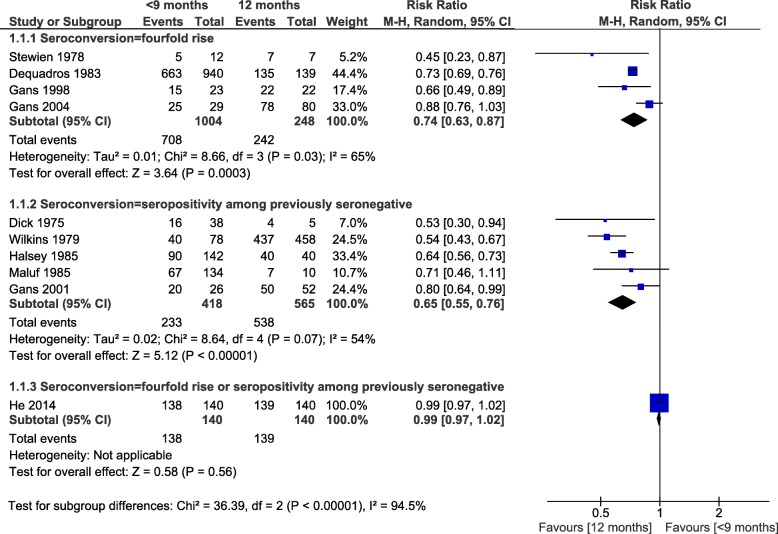
Age at first dose of measles-containing vaccine and seroconversion (< 9 months versus 12 months)

**Fig. 5:**
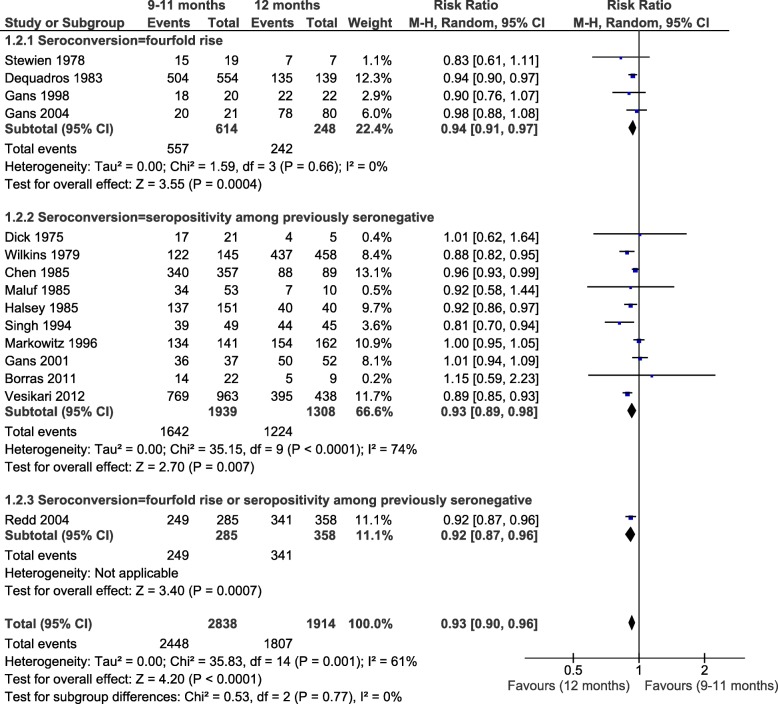
Age at first dose of measles-containing vaccine and seroconversion (9–11 months versus 12 months)

**Fig. 6:**
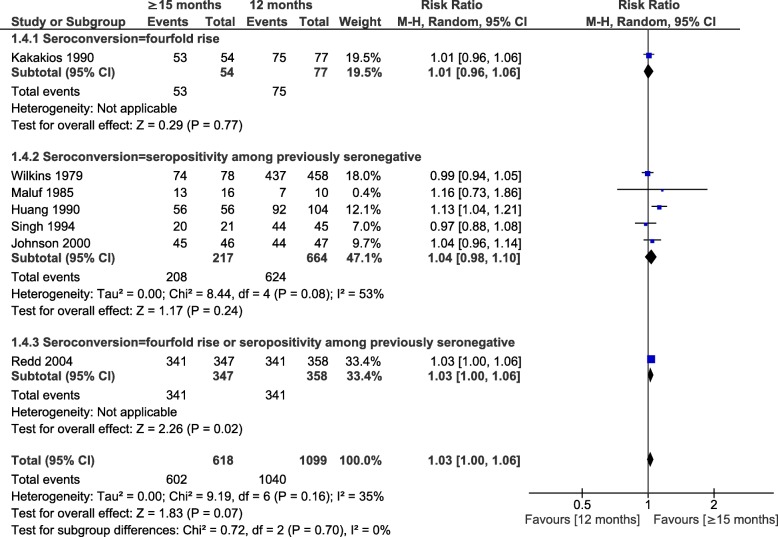
Age at first dose of measles-containing vaccine and seroconversion (≥15 months versus 12 months)

Seroconversion was lower in infants with MCV1 < 9 months than in children vaccinated at 12 months, with RRs of 0.74, 0.65 and 0.99 according to the definition of seroconversion (fourfold increase in titers (4 studies), seropositivity among seronegative pre-vaccination (5 studies) or both criteria (1 study)) (Fig. 4). The heterogeneity found in these comparisons was not explained by the predefined factors. The only RCT for this outcome reported 99% seroconversion for children vaccinated at 8 or 12 months (*n* = 280) (Additional file 6) [70]. Vaccination at 9–11 months versus 12 months yielded similar pooled RR of seroconversion for the 3 subgroup analyses, with an overall RR of 0.93 (95%CI: 0.90, 0.96; I^2^ = 61%; 15 studies) (Fig. 5). The two RCT included in this analysis (*n* = 1401 and 643) reported similar results: RR = 0.89 (95%CI = 0.85, 0.93) and RR = 0.92 (95%CI = 0.87, 0.96), respectively [8, 69]. Finally, MCV1 at ≥15 months induced a 3% higher seroconversion risk compared to 12 months (RR = 1.03, 95%CI: 1.00, 1.06; I^2^ = 35%; 7 studies), similar to the only RCT (*n* = 705) [8] (Fig. 6). Evidence from the experimental studies included in the one-dose serological analysis was rated as moderate, based on study design, consistency, low to moderate risk of bias, and dose response [131].

Sensitivity analyses including only studies at low risk of bias found similar results (Additional file 5).

Vaccination at 6 months induced lower seroconversion compared with 9 months (RR = 0.74, 95%CI: 0.68 to 0.82; I^2^ = 65%; 13 studies). However, there was significant heterogeneity for all sub-analyses, not explained by the predefined effect modifiers (Additional file 7).

Seropositivity after MCV2 was reported in 3 RCT and 4 Non-RCT (Additional file 2). The 3 RCT found high seropositivity in all two-dose recipients. It varied from 95 to 100% for first dose administered at 4–5, 8, 9, 11 or 12 months [69, 70, 74]. Only Vesikari et al. found a significant difference between MCV1 at 9 (95%) or 12 months (98%) [69]. A large non-RCT found increasing seropositivity with older age at MCV1, from 80% for MCV1 at 7–8 months to 96% for MCV1 at 10–11 months (*n* = 1111) [92]. Three non-RCT showed a similar seropositivity risk for MCV1 at 6 versus 9 months [6, 93] or inconsistent results [85] but the number of participants was small (Table 2).

**Table 2:** Seropositivity risk after two doses of measles vaccine by age at first vaccination

Author, year (strain) (ref)	N^**a**^	Seropositivity dfn^b^	Seropositivity risk by age at MVC1 [95%CI]					
			< 6	6	7–8	9	10–11	12
Soerensen, 1985 (Schwarz) [92]	1111	+			0.80 [0.77–0.82]	0.90 [0.83–0.98]	0.96 [0.90–1]	
McGraw, 1986 (Moraten) [85]	52	+			0.95 [0.85–1]	0.89 [0.76–1]		1.00 [−]
Gans, 2001 (Moraten) [6]	31	+		1.00 [−]		1.00 [−]		
Gans, 2004 (Moraten) [93]	50	+		0.86 [0.74–0.99]		0.90 [0.78–1]		
Vesikari, 2012 (Moraten) [69]	1364	+				0.95 [0.93–0.97]	0.98^c^ [0.96–0.99]	0.99 [0.97–1]
Martins, 2014 (EZ) [74]	655	+	0.97 [0.95–0.99]			0.99 [0.98–1]		
He, 2014 (Hu191) [70]	206	+			1.00^d^ [−]			1.00 [−]
Youwang, 2001 (Hu191) [132]	76	4-fold		0.40 [0.15–0.65]	0.52^e^ [0.40–0.65]			

Abbreviations: *CI* Confidence interval, *E-Z* Edmonston-Zagreb, *NR* Not reported, *MCV1* dose one of measles-containing vaccine

^a^ Number of cases for the calculation of the age effect

^b^ + = seropositivity among all participants; 4-fold = fourfold increase in antibody titers

^c^ 11 months

^d^ 8 months

^e^ 8 to 15 months

### Risk of bias across studies

Funnel plots did not show much asymmetry but most of the effect estimates were plotted close to the pooled measure, suggesting that publication bias might exist but did not have a major impact on our results (Additional file 8).

## Discussion

Overall, we found robust evidence that increased age at MCV1, from 6 to ≥15 months (by comparing 6 versus 9, 9–11 or < 12 versus 12–14 and 12–14 versus ≥15 months), improved antibody response and measles protection among one-dose recipients. Shortly after a second dose, serological studies showed high seropositivity regardless of age at administration of MCV1, with only two trials reporting lower seropositivity for MCV1 given at ≤9 months [69, 92]. Data from epidemic investigations suggested that less protection with earlier age at MCV1 could persist among two-dose recipients but this is based on only 5 reports, some of which included few cases [10, 38, 41]. In all analyses and for similar age categories, age at MCV1 was more strongly associated with measles risk than with seronegativity risk. This could be partly explained by the more controlled context of the experimental studies but it might also reflect secondary vaccine failures that would not be detected shortly after vaccination [133]. Serological studies have demonstrated that, in infants first vaccinated at 11–12 months, MCV1 induced high seroconversion rates but lower antibody titers than vaccination at 15 months [134]; post-second dose titers correlated highly with post-MCV1 titers, which may lead to a greater risk of waning immunity over time among poor responders [133–136].

In their systematic review, Uzicanin and Zimmerman reported a median VE of 77.0% (range: 26–99%) and 92.0% (range: 39–100%) after a single dose of MCV1 administered at 9–11 months or ≥ 12 months, respectively [17]. Lochlainn et al. reviewed immunogenicity, protection and safety for first measles vaccination below the age of 9 months [19]. Both papers summarized studies reporting VE and seroconversion for each age at vaccination. By reviewing studies that compared immunogenicity or VE at different ages at vaccination, we reduced the bias resulting from comparing VE at each age obtained from different studies, where exposition to disease, vaccine ascertainment and other factors could vary. Besides, by examining smaller age categories based on the most common schedule recommendations [14], our review refined the assessment of the role of age at MCV1 with respect to VE, serological results and two-dose protection. When there were few unvaccinated cases, as in school outbreak investigations, VE by age at vaccination could not be estimated or was difficult to interpret. We circumvented this problem by analyzing only vaccinated individuals and calculating their relative risks of measles for different ages at vaccination, thus minimizing the risk of bias associated with VE calculation from epidemics among highly vaccinated populations.

This review was limited by the quality of the epidemiological studies as only 27% were at low risk of bias. However, it is reassuring that the results of serological studies at low risk of bias were very much in line with those of epidemic investigations. Only 13% of the serological studies measured neutralizing antibodies, meaning that seroconversion might be underestimated when the protection threshold was quantified by less sensitive assays like HAI and ELISA [26–28, 137]. This would not change our conclusions if low levels of neutralizing antibodies were not protective in the medium term, as suggested by epidemiological results. As expected, data from the eligible studies were heterogeneous. Therefore, we restricted and stratified studies included in the quantitative analyses. We still found heterogeneity in some of the serological sub-analyses, which was not explained by region, year, vaccine strain or antibody assay. However, protection and immunogenicity pooled estimates showed the same direction of effect and were coherent, indicating that our results are robust despite the heterogeneity [30]. We could not use 12-month-old as the reference category for measles risk as most of the epidemiological studies presented together children vaccinated at 12 to 14 months. Even if the 12 months category would have been preferred to have data more comparable to immunological studies, vaccination at 12 to 14 months reflects the field reality of countries recommending measles vaccine at 12 months. Using it as a reference group was more informative than pooling the few studies that reported vaccination at 12 months. Finally, data on measles risk and seropositivity among two-dose recipients were limited and summary measures could not be calculated for these outcomes, highlighting the need for further evaluations of the effect of age at first dose after MCV2.

Over the last 20 years, two phenomena have influenced a progressive epidemiological transition with implications for policy concerning age at measles vaccination: first, infants have increasingly been born to vaccinated mothers and received less placental transferred maternal antibodies [138]; second, two-dose schedules became recommended worldwide [14]. Most of the studies included in this review had participants born to mothers with naturally acquired immunity. As no study presented information about maternal status (disease or vaccinated), it was not possible to do a sensitivity analysis on children born to vaccinated mothers who now represent the majority of newborns in countries with high vaccination coverage. However, age-differences in seroconversion among infants without detectable maternal antibodies before vaccination suggest that age-effect will still be present even in the situation of lower maternal antibody transfer [6].

All five WHO regions have set a target for measles elimination [2]. Although huge progress has been made in decreasing measles morbidity and mortality worldwide, only the Americas region has attained and maintained elimination [139]. Elimination requires achieving and maintaining the highest one-dose and two-dose vaccination coverage (95%) [2] while minimizing primary and secondary failures. Seropositivity data showed that primary vaccine failures are uncommon shortly after two doses even when MCV1 was administered at an age as early as 9 months [6, 69, 70, 74, 94]. However, these serological studies did not provide information about the risk of secondary vaccine failures. Epidemiological data concerning vaccine failures and sero-surveys suggest greater vulnerability than expected from seroconversion rates [12, 140–143]. The high correlation found between first- and second-dose antibody titers [97] and our post-first dose results suggest that age at MCV1 might still play a role in the current two-doses measles epidemiological context. While some researchers had advocated to decrease the age at first dose to 9 months in European countries [144, 145], our review rather supports to maintain the recommendation of MCV1’s administration after the first year of life and even consider vaccination at 15 months. In elimination and low-transmission jurisdictions the risk of measles for infants is very low [146] and a change of age at first vaccination would only increase vulnerability during few months (from 12 to 15 months of age for example) among infants of families accepting vaccination. Even considering this temporary increased risk in infants, older age at vaccination might slightly improve overall immunity in each cohort, which may be valuable if 94% immunity is necessary to maintain elimination and minimize secondary spread following importation [147]. The African and South-East Asian regions accounted for 85% of measles deaths in 2016 [139]. In these contexts, early vaccination at 9 months may still be warranted to protect infants from severe disease [32], even if the risk of vaccine failures might increase.

While two-dose vaccination coverage is the most critical factor in interrupting measles transmission [148], older age at first vaccination may be necessary to keep population immunity level high enough to maintain elimination.

## Conclusion

In children born to mothers who had measles, an earlier age at MCV1 decreases measles protection and immunogenicity after one dose and might also decrease protection after two doses of measles vaccine. For children born to vaccinated mothers, the effect of age at MCV1 in two-dose programs warrants further evaluation.

## Supplementary information

**Additional file 1.** Systematic review protocol. This document describes the protocol for the systematic review and meta-analysis.

**Additional file 2.** Table – Characteristics of included studies. This table describes the characteristics (first author, year, country, number of cases or participants, vaccine strain and study design) of the studies included in the review.

**Additional file 3.** Figure – Risk of bias of the observational studies. This figure represents the evaluation of the risk of bias (global and for each item) of the observational studies included in the analysis of measles protection.

**Additional file 4.** Figure – Risk of bias of the experimental studies. This figure represents the evaluation of the risk of bias (global and for each item) of the experimental studies (RCT and non-RCT) included in the immunogenicity analysis.

**Additional file 5.** Table – Sensitivity analysis with studies at low risk of bias. This table shows the effect estimate for each outcome including only the studies evaluated as having a low risk of bias.

**Additional file 6.** Table – Seroconversion after one dose of MCV by age at vaccination. This table describes the seroconversion risk by age at vaccination of all studies included in the one-dose immunogenicity analysis.

**Additional file 7.** Figure – Seroconversion after one dose of MCV: 6 versus 9 months. This figure is a forest plot of the meta-analysis comparing seroconversion after one dose of MCV at 6 months versus 9 months of age.

**Additional file 8.** Figure – Funnel plots. This figure represents the funnel plots for each outcome.

## Data Availability

Data sharing is not applicable to this article as no datasets were generated or analysed during the current study. All articles included in this review are publicly available.

## Abbreviations

AR: Attack rates
CF: Complement fixation test
CI: Confidence interval/s
ELISA: Enzyme immunoassay
HAI: Hemagglutination assay
MCV1: First dose of a measles-containing vaccine
MCV2: Second dose of a measles-containing vaccine
OR: Odds ratio
PRN: Plaque reduction neutralization test
RCT: Randomized controlled trial
RR: Risk ratio
VE: Vaccine effectiveness
WHO: World Health Organization
