# Supplementary material for: Effect of age at vaccination on the measles vaccine effectiveness and immunogenicity: systematic review and meta-analysis
Source: BMC Infect Dis. 2020 Mar 29;20:251. doi: 10.1186/s12879-020-4870-x (PMC7104533; doi:10.1186/s12879-020-4870-x)
Supplement: Supplementary file 1 — Additional file 1. Systematic review protocol. This document describes the protocol for the systematic review and meta-analysis. [file 12879_2020_4870_MOESM1_ESM.pdf]

Effect of age at vaccination on the measles vaccine immunogenicity and effectiveness: a systematic review protocol

Sara Carazo Perez, Marie-Noëlle Billard, Amélie Boutin, Gaston De Serres

Background

Measles-containing vaccine (MCV) effectiveness has been estimated to be 84 to 92.5% for one dose and 94% for two doses [1]. The immune response to MCV is affected by age at vaccination due to the interference of measles maternal antibodies and the immaturity of the immune system of younger infants [2-5]. Therefore, the age at vaccination needs to balance the risk of acquiring measles infection by infants with the decreased immunogenicity when measles vaccine was administered early in life [6]. The introduction of two-dose schedules aimed to seroconvert infants who did not respond to the first dose mainly due to low age at vaccination. [7]. However, epidemic investigations [8-10] and serological studies [11] have suggested that the effect of age at first vaccination could persist after two doses, with a lower protection among children vaccinated before 15 months. Although several studies have addressed the importance of the age at measles vaccination in the response to MCV [12, 13], no review has systematically summarized these data.

Objectives

To evaluate the effect of the age of administration of the first dose of MCV (MCV1) on the effectiveness and immunogenicity of measles vaccination with one and two doses, we have reviewed observational studies estimating vaccine effectiveness (VE) and/or measles attack rates (AR) by age at vaccination as well as experimental studies comparing seroconversion prevalence by age at vaccination.

Methods

Study design

We will conduct a systematic review of the literature following The Cochrane Handbook for Systematic Reviews of Interventions [14] methodological recommendations, and we will report our results according to the Preferred Reporting Items for Systematic Reviews and Meta-Analyses (PRISMA) statement [15].

Eligibility criteria

The inclusion criteria are based on the PICOS (population, interventions, comparisons, outcomes and study design) framework (Table 1).

To evaluate measles vaccine effectiveness by age at vaccination, cohort and case-control studies are included if at least 80% of participants were first vaccinated with a further attenuated live measles-containing vaccine without concomitant administration of anti-measles immunoglobulin during the first two years of life and at least two weeks before outbreak onset. The two outcomes of interest are the vaccine effectiveness and the measles attack rates by age at vaccination.

To assess the effect of age on the antibody response, randomized control trials (RCT) and quasi-experimental studies are included if the first dose of a further attenuated live standard dose of MCV was administered intramuscular or subcutaneously before the age of two years. Studies of killed and high titre vaccines are excluded, as well as those administered by aerosol or intradermal. Studies targeting populations with special characteristics that could affect their response to vaccination, like immunosuppressed or malnourished children [16], are excluded, but not those reporting less than 5% of acute malnutrition in the general population of children [17]. When different vaccine strains were administered in one study, several results will be extracted to compare children receiving same strain at different age. The outcomes to be examined are the seroconversion after MCV1 and the seropositivity after MCV2.

All studies published in English, French, Spanish or Portuguese are eligible. Table 2 summarizes the inclusion criteria.

Information sources

Studies will be identified by a systematic search of the databases PubMed/MEDLINE, Embase, Web of Science and Cochrane from 1964, when the first measles vaccine was licensed, up to May 2017. Additionally, references of selected articles and key published reviews [1, 18-20] will be also hand searched.

Search strategy

The terms included in the MEDLINE search are: Measles Vaccine, Measles-Mumps-Rubella Vaccine, Measles/prevention and control, Vaccination, Measles Mumps Rubella Varicella vaccin*, MMR, MMRV, Vaccine effectiveness, Efficacy, Epidemic, Outbreak, Treatment failure, Vaccine failure, Antibod*, Serologic Tests, Seroconversion, Immunogenicity, Age at vaccination, Age at immunization and Age factor, all combined with the appropriate connectors. The search strategy, validated by a professional librarian, will be subsequently

adapted to the other databases. Results and detailed search strategy for each database is presented in Table 3.

Study Selection

After elimination of duplicates using the EndNote software and manually completed, two reviewers (SCP and MNB) will independently select the studies based on the described criteria. An initial pilot phase based on 25 references will facilitate clarification of the inclusion criteria. The process will followe the standard stages, with a first screening based on title and abstract and the final inclusion decided after revision of the full text. All disagreements will be solved by consensus or consultation of a third party (GDS). The main reason for exclusion will be recorded during the full text examination.

Data Collection Process

A data extraction form will be developed including the variables of interest regarding: (1) the study's characteristics (author, year of publication, year of epidemic/study, country, design, language, and funding), (2) the population (sex, nutrition status, immunization status of cases and non-cases, presence of maternal antibodies pre-immunization), (3) the intervention (measles vaccine strain, age at vaccination, number of doses, interval between doses, vaccine status ascertainment, and immunization context) and (4) the outcome (measles case definition, seroconversion definition, assay for antibody measurement, attack rates, seroconversion and seropositivity prevalence). An adapted form will be used for each analysis (immunogenicity or effectiveness). The forms will be tested by two reviewers (SCP and MNB) with three articles and adapted consequently.

One of the authors (SCP or MNB) will collect the data from the studies, which will be checked by a second reviewer (SCP or MNB). When missing or inaccurate information, authors of the articles will be contacted. If not reported, but enough data allow the estimation of vaccine effectiveness or seroconversion prevalence by age at vaccination, the calculation will be done by the reviewers. Age categories with less than 10 subjects will be grouped before inclusion in the meta-analysis in order to increase statistical power.

Data items

VE is defined as the protection against measles conferred by the vaccine in field conditions [21]. It can be calculated comparing the attack rates among vaccinated and non-vaccinated or comparing the vaccination status of cases and non-cases during measles epidemics [22, 23]. Measles cases are defined by clinical, epidemiological and/or immunological criteria. Attenuated or non-classical measles cases will be included only if confirmed by laboratory [6].

Seroconversion is defined, according to the study, as the presence of measles antibodies in individuals with previously undetectable titers, the fourfold increase in their concentration or both, while seropositivity corresponds to an antibody concentration higher than the protective threshold [24]. Assays to measure antibody levels include the enzyme immunoassay (ELISA), hemagglutination (HAI), plaque reduction neutralization (PRN) or complement fixation (CF) tests [25, 26].

Risk of bias of individual studies

Two reviewers (SCP and MNB) will independently evaluate the risk of bias of each outcome in each study.

For the assessment of the observational studies, a scale will be adapted from the NICE public health guidance [27] to evaluate the study representativeness, the selection process, the comparability of the groups, the vaccination status ascertainment and the outcome definition and completeness [23] (Table 4).

The Cochrane collaboration tool [14] will be adapted to evaluate the risk of bias in clinical trials. The representativeness, the risk of selection bias, performance bias, comparability, attrition bias, information bias and reporting bias will be evaluated for each outcome. As the presence of maternal antibodies is one of the main factors influencing the response to measles vaccine by age at vaccination, experimental studies excluding children with maternal antibodies will be considered of low quality in the evaluation of the comparability between groups (Table 5).

Summary measures and synthesis of results

In all analysis, one and two dose recipients will be analyzed separately.

In the qualitative summary of the field protection conferred by MCV1 and MCV2, we will report VE estimations for each age group.

All quantitative meta-analysis for the defined outcomes will be performed using random effect models. A visual examination of the forest plot and a statistic measure of heterogeneity (I^2), generated by RevMan 5.3, will be used to evaluate homogeneity between studies. An I^2 statistic of >50% will be interpreted as significant heterogeneity [28], which will be therefore explored according to a priori identified factors, known to be potential effect modifiers.

Based on study design, risk ratios (RR) or odds ratios (OR) of measles by age at vaccination will be calculated when possible. Meta-analysis will be performed to pool RR/OR of children first vaccinated before 9 months, between 9 to 11 months and at 15 months or older, all

compared with the category containing those vaccinated at 12 months. These age categories were chosen based on current vaccination policies [29-31]. In case of significant heterogeneity, subgroup analysis are planned to evaluate the modifying effect of the measles case definition, the WHO world region and the year of epidemic (before or after 1985).

For the immunogenicity analysis, seroconversion or seropositivity prevalence after MCV1 or MCV2 will be presented by age at vaccination. The effect of age at vaccination will be summarized computing the pooled prevalence ratio (PR) of seroconversion/seropositivity that compares the same age categories previously described. Only studies with a standard definition of seroconversion (fourfold increase with or without adjustment for antibody decay, seropositivity among previously seronegative or both) will be included in the quantitative pooled analysis. The pooled PR will be calculated separately by seroconversion definition and the overall measure of association will be presented only if the test for subgroup differences is non-significant. To increase the power of these meta-analyses, all studies allowing calculation of different seroconversion definitions will be included in each subgroup analysis. In case of heterogeneity, further stratified analyses will be conducted to test the modifying effect of the vaccine strain, the assay to quantify antibody titers and the year of study. Studies exclusively presenting the seropositivity after MVC1 or using an alternative definition of seroconversion will be presented only in the qualitative results.

Risk of bias across studies

The publication bias will be visually evaluated for each outcome by examination of a funnel plot.

Additional analysis

A pre-specified sensitivity analyses for studies with low risk of bias and for RCTs will be conducted for all outcomes when appropriate.

Tables

Table 1. Research question structured according to PICOS

Population	Participants first vaccinated against measles below 2 years of age
Intervention	Immunization with one or two doses of further attenuated live measles-containing vaccine
Comparator	4 comparison groups by age at first vaccination: less than 9 months, 9-11 months, 12 or 12-14 months and 15 months or older.<br>Vaccination at 12-14 months as reference category.
Primary outcomes	Vaccine effectiveness by age at first vaccination<br>Measles risk ratio or odds ratio by age at first vaccination<br>Seroconversion or seropositivity prevalence ratio by age at first vaccination
Study design	Cohort, case-controls and randomized trials

Table 2. Inclusion criteria for article selection

Effectiveness analysis	Immunogenicity analysis
Vaccination with one or two doses of a further attenuated live measles-containing vaccine without IgG co-administration (≥80% of at-risk population)	Intramuscular or subcutaneous vaccination with one or two doses of a further attenuated live measles-containing vaccine
First dose of measles vaccine administered before the age of two years	First dose of measles vaccine administered before the age of two years
Vaccine effectiveness or attack rates reported by age at first vaccination	Antibody response reported by age at first vaccination
Same number of doses for the compared groups	Same intervention with respect to the number of doses and measles strain vaccine
Observational studies: Cohort, case-control	Experimental studies: Randomized and non-randomized trials
Publication in English, French, Spanish or Portuguese	Publication in English, French, Spanish or Portuguese

Table 3: Search strategies in MEDLINE, Web of Science, Embase and Cochrane databases

#	**A. MEDLINE** [Period: 1964 to 8, May 2017]	**Results**
1	**Intervention**: [vaccination with a measles-containing vaccine]	
	"Measles Vaccine"[Mesh] OR "Measles-Mumps-Rubella Vaccine"[Mesh] OR ("Measles/prevention and control"[Mesh:NoExp] AND "Vaccination"[Mesh]) OR Measles vaccin* [TIAB] OR (Measles Mumps Rubella vaccin*[TIAB]) OR (Mumps Measles Rubella vaccin*[TIAB]) OR (Measles Mumps Rubella Varicella vaccin*[TIAB]) OR (MMR vaccin*[TIAB]) OR (MMRV vaccin*[TIAB]) OR Priorix*[TIAB] OR (Triviraten[TIAB] AND Berna[TIAB]) OR Trimovax[TIAB] OR Virivac[TIAB] OR Pluserix[TIAB] OR ProQuad[TIAB]	9,834
2	**Outcome**: [vaccine efficacy or immunogenicity]	
	"Antibodies, Viral"[Mesh:NoExp] OR "Serologic Tests"[Mesh] OR Seroconversion[TIAB] OR Immunogenicity[TIAB] OR Serological[TIAB] OR Antibod*[TIAB] OR "Vaccine effectiveness"[TIAB] OR Efficacy[TIAB] OR Epidemic[TIAB] OR Outbreak[TIAB] OR "Treatment failure"[Mesh] OR "Vaccine failure"[TIAB]	1,672,118
3	**Comparison**: [age at first vaccination]	
	“Age Factors”[Mesh] OR "Age factor*" [TIAB] OR “Age at vaccination” OR “Age at immunization” OR “Age at first vaccination”	455,817
4	Studies in humans	
	Animals [Mesh] NOT Humans [Mesh]	
5	#1 AND #2 AND #3 NOT #4	624

#	**B. WEB OF SCIENCE** [Period: 1964 to 8, May 2017]	**Results**
1	**Intervention**: [vaccination with a measles-containing vaccine]	
	TS=('measles vaccine' OR (measles NEAR/3 (vaccin* OR immunization)) OR mmr OR mmrv OR priorix* OR 'triviraten berna' OR trimovax OR virivac OR pluserix OR proquad OR immoravax OR 'moruviraten berna' OR tresivac)	14,642
2	**Outcome**: [vaccine efficacy or immunogenicity]	
	TS=('measles antibody' OR serodiagnosis OR 'drug efficacy' OR ‘vaccine effectiveness’ OR Immunogenicity OR Antibod* OR epidemic OR outbreak OR 'treatment failure' OR (vaccine NEAR/2 failure))	1,331,874
3	**Comparison**: [age at first vaccination]	
	TS=(age OR 'age group' OR 'age factor')	2,431,669
4	#1 AND #2 AND #3	**1,676**

#	**C. EMBASE** [Period: 1964 to 8, May 2017]	**Results**
1	**Intervention**: [vaccination with a measles-containing vaccine]	
	'measles vaccine':de,ab,ti OR (measles NEAR/4 (vaccin* OR immunization)):ab,ti OR (mmr NEXT/2 (vaccin* OR immunization)):ab,ti OR (mmrv NEXT/2 (vaccin* OR immunization)):ab,ti OR priorix*:ab,ti OR 'triviraten berna':ab,ti OR trimovax:ab,ti OR virivac:ab,ti OR pluserix:ab,ti OR proquad:ab,ti OR 'm r vax':ab,ti OR immoravax:ab,ti OR 'moruviraten berna':ab,ti OR tresivac:ab,ti	13,447
2	**Outcome**: [vaccine efficacy or immunogenicity]	
	'measles antibody'/de OR 'serodiagnosis'/de OR 'drug efficacy'/de OR ‘vaccine effectiveness’:ti,ab OR Immunogenicity:ti,ab OR Antibod*:ti,ab OR 'epidemic':de,ti,ab OR outbreak:ti,ab OR 'treatment failure'/exp OR (vaccine AND failure):ab,ti	2,003,591

3	**Comparison**: [age at first vaccination]	
	'age'/exp OR 'age group':ti,ab OR 'age factor':de,ti,ab OR ‘age at vaccination’:de,ti,ab	853,728
4	Studies in humans	
	'animal'/exp NOT 'human'/exp	
5	#1 AND #2 AND #3 NOT #4	**549**

#	**D. COCHRANE** [Period: 1964 to 8, May 2017]	**Results**
1	**Intervention**: [vaccination with a measles-containing vaccine]*	
	MeSH descriptor:[Measles Vaccine] explode all trees OR (measles NEAR/4 (vaccin* OR immunization)):ab,ti,kw OR mmr:ab,ti,kw OR mmrv:ab,ti,kw OR priorix*:ab,ti,kw OR 'triviraten berna':ab,ti,kw OR trimovax:ab,ti,kw OR virivac:ab,ti,kw OR pluserix:ab,ti,kw OR proquad:ab,ti,kw OR 'm r vax':ab,ti,kw OR immoravax:ab,ti,kw OR 'moruviraten berna':ab,ti,kw OR tresivac:ab,ti,kw	869
2	**Outcome**: [vaccine efficacy or immunogenicity]	
	MeSH descriptor:[Antibodies, Viral] this term only OR MeSH descriptor:[Serologic Tests] explode all trees OR ‘vaccine effectiveness’:ti,ab,kw OR Immunogenicity:ti,ab,kw OR Antibod*:ti,ab,kw or seroconversion:ti,ab,kw OR epidemic:ti,ab,kw OR outbreak:ti,ab,kw OR MeSH descriptor:[Treatment Failure] explode all trees OR (vaccine AND failure):ab,ti,kw	34,492
3	Studies in humans	
	MeSH descriptor:[Animals] explode all trees	
4	#1 AND #2 NOT #3	**412**

Table 4: Tool to evaluate the risk of bias in observational studies (vaccine effectiveness analysis)

EVALUATION OF RISK OF BIAS: Observational studies*			
**CODE**: ++: the risk of bias has been minimised<br>+: answer is not clear or not all potential sources of bias have been addressed<br>-: significant sources of bias may persist<br>NR: not reported<br>NA: not applicable			
**SECTION**	**ITEM**	**LABEL**	**COMMENTS** (items to be checked)
**Study design**	Design	Study design	Study design based on the NICE's public health guidance
**Representativeness**	Source population	Is the source population/area well described?	Description of country, setting, location and population demographics.
**Representativeness**	Eligible population	Is the eligible population/area representative of the source population/area?	-Recruitment well defined<br>-Eligible population representative of the source (school epidemics considered representative of that population)
**Representativeness**	Participants	Do the selected participants/areas represent the eligible population/area?	-Method of selection of participants: cases representative of the epidemic (++) or hospitalised cases (-)<br>-% of selected who agree to participate:<br>(++) ≥90% participation<br>(+) 60-90% participation<br>(-) <60% participation<br>-Inclusion/ exclusion criteria
**Representativeness**	Representativeness	Global score	Based on the 3 previous items
**Selection**	Selection of the comparison group	Is comparison group drawn from the same community as the exposed cohort?<br>Are controls selected from the same source than cases?	Risk of selection bias based on the selection of comparison group (cohort study) or control group (case-control study).
**Selection**	Response rate	Is there the same rate of non-response for both groups?	Non-response rate for cases and controls (case-control studies) /population at risk (retrospective cohorts)
**Selection**	Selection	Global score	Based on the 2 previous items
**Comparability**	Comparability	Are the groups comparable by design or analysis? Potential confounders:<br>- Time since vaccination<br>- Malnutrition status<br>- Vaccine strain and potential use of killed vaccine or IgG<br>- Exposure to measles<br>- Affected by control measures	Potential confounders:<br>(++): controlled or not associated to age at vaccination<br>(+): not clear or potential confounding still existing<br>(-): probable association to age at vaccination and not controlled for it

**Exposure**	Vaccination ascertainment	Has the vaccination status been ascertained vaccine record?	Vaccination status ascertained by:<br>(++): vaccine record<br>(+): school record<br>(-): interview/self-reported
**Exposure**	Vaccination ascertainment for the comparison group	Has the same method been used for both groups to ascertain the vaccination status?	-Method to ascertain vaccination status similar for cases and controls /exposed and non-exposed: (++) yes; (-) non;<br>-Number with unknown vaccination status in each group
**Exposure**	Measles history	Is there a demonstration of no history of measles among controls /non-exposed?	(++): Active questioning on measles history among controls / non-exposed group?<br>(+): not reported but low possibility of previous measles history<br>(-): not reported and high possibility of previous measles history
**Exposure**	Exposure	Global score	Based on the 3 previous items
**Outcome**	Case ascertainment	Have the measles cases been well defined?	(++): cases defined by laboratory or epidemiological link to a confirmed case<br>(+): cases defined by clinical signs or epidemiological link<br>(-): cases defined by official records, nurse diagnosis without case definition or questionnaire to families
**Outcome**	Incomplete outcome	Has the outcome been completely reported?	(++): active search of all cases identified<br>(+): no active search<br>(-): very probably some cases not included
**Outcome**	Outcome	Global score	Based on the 2 previous items

*Adapted from the NICE public health guidance

Table 5: Tool to evaluate the risk of bias in experimental studies (immunogenicity analysis)

EVALUATION OF RISK OF BIAS: Experimental studies*			
**CODE**: ++: the risk of bias has been minimised<br>+: answer is not clear or not all potential sources of bias have been addressed<br>-: significant sources of bias may persist<br>NR: not reported<br>NA: not applicable			
**SECTION**	**ITEM**	**LABEL**	**COMMENTS** (items to be checked)
**Study design**	Design	Study design	Study design based on the NICE's public health guidance
**Representative ness**	Source population	Is the source population/area well described?	Description of country, setting, location and population demographics.
**Representative ness**	Eligible population	Is the eligible population/area representative of the source population/area?	-Recruitment well defined<br>-Eligible population representative of the source
**Representative ness**	Participants	Do the selected participants/areas represent the eligible population/area?	-Method of selection of participants<br>-% of selected who agree to participate<br>-Inclusion/ exclusion criteria
**Representative ness**	Representati-veness	Global score	Based on the 3 previous items
**Selection bias**	Randomiza-tion	Random sequence generation	Method used to generate the allocation sequence in sufficient detail to allow an assessment of whether it should produce comparable groups (for RCT)
**Selection bias**	Baseline	Selection of non-randomized groups	Differences in compared groups due to differential selection (sex, maternal ab, malnutrition, measles history)<br>(++): exclusion if measles history, not differences on sex /malnutrition distribution<br>(-): differential selection is probable
**Selection bias**	Selection	Global score	Based on the 2 previous items
**Performance bias**	Blinding of participants	Blinding of participants and personnel	Measures to blind study participants and personnel from knowledge of which intervention a participant received (for RCT)<br>(++): Blindness or no impact on results
**Comparability**	Maternal antibodies	- Stratification or regression for the presence of maternal antibodies	Maternal antibodies have been measured and taken into account in the analysis.<br>(++): all (with and without maternal ab) included or both measures given<br>(-): restriction to seronegative

**Comparability**	Control for other confounders	Potential confounders:<br>- Malnutrition status<br>- Measles exposition pre or post-vaccination<br>- Time from vaccination to sampling<br>- Interval between doses	(++): adjustment if necessary<br>(+): partial adjustment<br>(-): bias potential due to not adjustment when potential confounding existing
**Comparability**	Confounding	Global score	Based on the 2 previous items
**Attrition bias**	Incomplete outcome	Incomplete outcome data	Completeness of outcome data, including attrition and exclusions from the analysis: if reported, numbers in each group and reasons.<br>(++): number and reasons reported<br>(+): no reported the reasons
**Information bias**	Intervention	Intervention	All participants received the same intervention: vaccine strain and potency
**Information bias**	Outcome	Lab method	(++): PRN<br>(+): other (ELISA, HAI)<br>(-): dry pad
**Information bias**	Information bias	Global score	Based on the 2 previous items
**Reporting bias**	Selective reporting	Selective reporting	Possibility of selective outcome reporting.

*Adapted from the Cochrane collaboration tool

References

[1] Uzicanin A, Zimmerman L. Field effectiveness of live attenuated measles-containing vaccines: a review of published literature. The Journal of infectious diseases. 2011;204 Suppl:S133-48.
[2] Gans H, Yasukawa L, Rinki M, DeHovitz R, Forghani B, Beeler J, et al. Immune responses to measles and mumps vaccination of infants at 6, 9, and 12 months. The Journal of infectious diseases. 2001;184:817-26.
[3] Klinge J, Lugauer S, Korn K, Heininger U, Stehr K. Comparison of immunogenicity and reactogenicity of a measles, mumps and rubella (MMR) vaccine in German children vaccinated at 9-11, 12-14 or 15-17 months of age. Vaccine. 2000;18:3134-40.
[4] Redd SC, King GE, Heath JL, Forghani B, Bellini WJ, Markowitz LE. Comparison of vaccination with measles-mumps-rubella vaccine at 9, 12, and 15 months of age. The Journal of infectious diseases. 2004;189:S116-22.
[5] Leuridan E, Van Damme P. Passive transmission and persistence of naturally acquired or vaccine-induced maternal antibodies against measles in newborns. Vaccine. 2007;25:6296-304.
[6] Strebel PM, Papania MJ, Fiebelkorn AP, Halsey N. Measles vaccine. In: Plotkin SA, Orenstain WA, Offit PA, editors. Vaccines. 6th ed. China: Elsevier Saunders; 2013.
[7] World Health Organization. Global measles and rubella strategic plan: 2012-2020. Geneva: WHO; 2012.
[8] De Serres G, Boulianne N, Meyer F, Ward BJ. Measles vaccine efficacy during an outbreak in a highly vaccinated population: incremental increase in protection with age at vaccination up to 18 months. Epidemiology and infection. 1995;115:315-23.
[9] Paunio M, Peltola H, Valle M, Davidkin I, Virtanen M, Heinonen OP. Twice vaccinated recipients are better protected against epidemic measles than are single dose recipients of measles containing vaccine. Journal of epidemiology and community health. 1999;53:173-8.
[10] Defay F, De Serres G, Skowronski DM, Boulianne N, Ouakki M, Landry M, et al. Measles in children vaccinated with 2 doses of MMR. Pediatrics. 2013;132:e1126-33.
[11] Poethko-Muller C, Mankertz A. Sero-epidemiology of measles-specific IgG antibodies and predictive factors for low or missing titres in a German population-based cross-sectional study in children and adolescents (KiGGS). Vaccine. 2011;29:7949-59.
[12] Vesikari L, Becker T, Gajdos V, Fiquet A, Thomas S, Richard P, et al. Immunogenicity and safety of a two-dose regimen of a combined measles, mumps, rubella and varicella live vaccine (ProQuad(®)) in infants from 9 months of age. Vaccine. 2012;30:3082-9.
[13] Gans HA, Yasukawa LL, Alderson A, Rinki M, DeHovitz R, Beeler J, et al. Humoral and cell-mediated immune responses to an early 2-dose measles vaccination regimen in the United States. Journal of Infectious Diseases. 2004;190:83-90.
[14] Cochrane handbook for systematic reviews of interventions. In: Higgins JPT, Green S, editors.: Cochrane Collaboration; 2011.
[15] Liberati A, Altman DG, Tetzlaff J, Mulrow C, Gotzsche PC, Ioannidis JP, et al. The PRISMA statement for reporting systematic reviews and meta-analyses of studies that evaluate health care interventions: explanation and elaboration. J Clin Epidemiol. 2009;62:e1-34.
[16] Waibale P, Bowlin SJ, Mortimer EA, Jr., Whalen C. The effect of human immunodeficiency virus-1 infection and stunting on measles immunoglobulin-G levels in children vaccinated against measles in Uganda. International journal of epidemiology. 1999;28:341-6.

[17] World Health Organization. Global Database on Child Growth and Malnutrition. WHO; 1997.
[18] Lochlainn LN, de Gier B, van der Maas NAT, Rots N, Van Binnendijk RS, De Melker HE, et al. Measles vaccination below 9 months of age: Systematic literature review and meta-analysis of effects and safety. In: Control CfID, editor. Bilthoven, the Netherlands2015. p. 109.
[19] Demicheli V, Rivetti A, Debalini MG, Di Pietrantonj C. Vaccines for measles, mumps and rubella in children. The Cochrane database of systematic reviews. 2012;2:CD004407-CD.
[20] Moss W, Scott S. The Immunological Basis for Immunization Series. Module 7: Measles. Update 2009. Geneva: WHO; 2009. p. 62.
[21] Weinberg GA, Szilagyi PG. Vaccine epidemiology: efficacy, effectiveness, and the translational research roadmap. The Journal of infectious diseases. 2010;201:1607-10.
[22] Orenstein WA, Bernier RH, Dondero TJ, Hinman AR, Marks JS, Bart KJ, et al. Field evaluation of vaccine efficacy. Bulletin of the World Health Organization. 1985;63:1055-68.
[23] Orenstein WA, Bernier RH, Hinman AR. Assessing vaccine efficacy in the field. Further observations. Epidemiologic reviews. 1988;10:212-41.
[24] Chen RT, Markowitz LE, Albrecht P, Stewart JA, Mofenson LM, Preblud SR, et al. Measles antibody: reevaluation of protective titers. The Journal of infectious diseases. 1990;162:1036-42.
[25] Neumann PW, Weber JM, Jessamine aG, O'Shaughnessy MV. Comparison of measles antihemolysin test, enzyme-linked immunosorbent assay, and hemagglutination inhibition test with neutralization test for determination of immune status. Journal of clinical microbiology. 1985;22:296-8.
[26] Ratnam S, Gadag V, West R, Burris J, Oates E, Stead F, et al. Comparison of commercial enzyme immunoassay kits with plaque reduction neutralization test for detection of measles virus antibody. Journal of clinical microbiology. 1995;33:811-5.
[27] NICE. Methods for the development of NICE public health guidance (third edition). 2012. p. 214-186.
[28] Higgins JP, Thompson SG, Deeks JJ, Altman DG. Measuring inconsistency in meta-analyses. BMJ (Clinical research ed). 2003;327:557-60.
[29] World Health Organization. Measles vaccines: WHO position paper, April 2017 - Recommendations. Vaccine. 2017.
[30] Strikas RA. Advisory committee on immunization practices recommended immunization schedules for persons aged 0 through 18 years--United States, 2015. MMWR Morbidity and mortality weekly report. 2015;64:93-4.
[31] European Centre for Disease Prevention and Control. Vaccine Schedule. 2015.
