# Supplementary material for: Effect of age at vaccination on the measles vaccine effectiveness and immunogenicity: systematic review and meta-analysis
Source: BMC Infect Dis. 2020 Mar 29;20:251. doi: 10.1186/s12879-020-4870-x (PMC7104533; doi:10.1186/s12879-020-4870-x)
Supplement: Supplementary file 2 — Additional file 2. Table – Characteristics of included studies. This table describes the characteristics (first author, year, country, number of cases or participants, vaccine strain and study design) of the studies included in the review. [file 12879_2020_4870_MOESM2_ESM.docx]

**Supplementary Table 1. Characteristics of the included studies**

| **A. MEASLES PROTECTION ANALYSIS– Observational studies** | | | | | | | | | |
| --- | --- | --- | --- | --- | --- | --- | --- | --- | --- |
| **First author,**  **epidemic year (ref)** | **Country** | **Cases in epidemic** | **Study design** | **Vaccine strain** | **Vaccination document^1^** | **Case definition: lab or epidemiol. link^2^** | **VE^3^ (%)** | **Doses** | |
|  |  |  |  |  |  |  |  | **1** | **2** |
| Shelton, 1976 [52] | USA | 294 | Case-control | NR | ✓ | NR | NA | ✓ | ✗ |
| McIntyre, 1977 [31] | Marshall Islands | 340 | Cohort | LFA | ✓ | Partially | 83.5 | ✓ | ✗ |
| Judelsohn, 1978 [53] | USA | 203 | Cohort | NR | ✓ | ✗ | NA | ✓ | ✗ |
| Faust, 1978 [54] | USA | 350 | Cohort | NR | ✓ | ✗ | NA | ✓ | ✗ |
| Lopes, 1979 [55] | Brazil | 19 | Cohort | LFA | ✓ | ✗ | NA | ✓ | ✗ |
| Aaby, 1980 [32] | Guinea-Bissau | NR | Cohort | Schwarz | Partially | ✗ | 71.4 | ✓ | ✗ |
| Hull, 1981 [33] | Gambia | 146 | Cohort | Moraten | ✓ | ✗ | 77.7 | ✓ | ✗ |
| Wassilak, 1981 [56] | USA | 156 | Case-control | NR | ✓ | Partially | NA | ✓ | ✗ |
| Anonymous, 1983 [34] | Brazil | NR | Cohort | NR | ✗ | NR | NA | ✓ | ✗ |
| Hull, 1984 [57] | USA | 76 | Case-control | Moraten | ✓ | ✓ | NA | ✓ | ✗ |
| Nkowane, 1984 [35] | USA | 27 | Cohort | NR | ✓ | ✗ | 94.4 | ✓ | ✗ |
| McCombie, 1985 [36] | USA | 225 | Cohort | NR | ✓ | Partially | 41.4 / 58.3 | ✓ | ✓ |
| Davis, 1985 [37] | USA | 137 | Cohort | NR | ✓ | ✓ | 96.9 | ✓ | ✗ |
| Chen, 1985 [58] | USA | 115 | Case-control | NR | ✓ | Partially | NA | ✓ | ✗ |
| Mast, 1986 [59] | USA | 219 | Case-control | NR | ✓ | ✓ | 93.2 | ✓ | ✗ |
| Robertson, 1986 [38] | USA | 284 | Cohort | NR | ✓ | ✓ | 81.9 / 94.1 | ✓ | ✓ |
| George, 1986 [39] | India | NR | Cohort | Schwarz, Moraten | ✓ | ✗ | 52.9 | ✓ | ✗ |
| Sharma 1987 [40] | India | 132 | Cohort | NR | Partially | ✗ | 53.0 | ✓ | ✗ |
| Hutchins, 1987 [60] | USA | 323 | Cohort | NR | ✓ | ✓ | NA | ✓ | ✗ |
| Hersh, 1987 [41] | USA | 84 | Cohort | NR | ✓ | Partially | 91.9 / 96.9 | ✓ | ✓ |
| Agocs, 1988 [61] | Hungary | 17938 | Cohort | Leningrad 16 | ✓ | ✗ | 73.2 | ✓ | ✗ |
| Lee, 1988 [47] | Taiwan | 2459 | Cohort | NR | ✓ | ✓ | 79.7 | ✓ | ✗ |
| Paunio, 1988 [10] | Finland | 1748 | Case-control | Schwarz, Moraten | ✓ | ✓ | NA | ✓ | ✓ |
| Chawla, 1989 [3] | India | 176 | Cohort | NR | ✓ | ✗ | 86.1 | ✓ | ✗ |
| Ng, 1989 [62] | Canada | 38 | Cohort | Moraten | ✓ | ✓ | NA | ✓ | ✗ |
| De Serres, 1989 [63] | Canada | 1363 | Case-control | Moraten | ✓ | Partially | NA | ✓ | ✗ |
| Rivest, 1989 [4] | Canada | 10184 | Case-control | LFA | ✓ | Partially | 95.9 | ✓ | ✗ |
| De la Puente, 1990 [43] | Spain | 95 | Cohort | NR | ✓ | ✗ | 66.4 | ✓ | ✗ |
| Malfait, 1990 [44] | Niger | 13578 | Cohort | Schwarz | ✓ | ✗ | 89.3 | ✓ | ✗ |
| De Serres, 1990 [64] | Canada | 11427 | Case-control | NR | ✓ | ✗ | NA | ✓ | ✓ |
| Murray, 1990 [5] | Pakistan | NR | Cohort | Schwarz | ✓ | ✗ | 84.3 | ✓ | ✗ |
| Coetzee, 1992 [45] | South Africa | 757 | Cohort | Schwarz | ✗ | Partially | 78.6 | ✓ | ✗ |
| McDonnell, 1993 [46] | Australia | 900 | Case-control | NR | ✓ | ✗ | 95.3 | ✓ | ✗ |
| Patel, 1994 [65] | Australia | 258 | Cohort | NR | ✓ | ✓ | NA | ✓ | ✗ |
| Lee, 1994 [47] | Taiwan | 49 | Cohort | NR | ✓ | ✓ | 85.8 | ✓ | ✗ |
| Sutcliffe, 1995 [66] | Canada | 800 | Cohort | NR | ✓ | Partially | -238.1 | ✓ | ✗ |
| Kaninda, 1995 [48] | Niger | 13892 | Cohort | Schwarz | ✓ | ✗ | 93.5 | ✓ | ✗ |
| Kotb 1995 [49] | Egypt | 230 | Case-control | NR | ✓ | ✗ | 53.4 | ✓ | ✗ |
| Hennessey, 1996 [50] | Romania | 32915 | Cohort | Schwarz | ✓ | ✗ | 88.6 | ✓ | ✗ |
| John, 1999 [51] | India | NR | Cohort | LFA | NR | ✗ | 29.6 | ✓ | ✗ |
| Defay, 2011 [11] | Canada | 725 | Case-control | Moraten | ✓ | ✓ | NA | ✗ | ✓ |

| **B. IMMUNOGENICITY ANALYSIS – Experimental or quasi-experimental studies** | | | | | | | |
| --- | --- | --- | --- | --- | --- | --- | --- |
| **First author, year of publication (ref)** | **Country** | **N^4^** | **Study design** | **Vaccine strain** | **Lab assay (+)^5^** | **Doses** | |
|  |  |  |  |  |  | **1** | **2** |
| Miller, 1967 [102] | Honduras | 278 | Before-after | Schwarz | HAI (1:5) | ✓ | ✗ |
| Ruben, 1973 [103] | Nigeria | 344 | Before-after | LFA | HAI (1:5) | ✓ | ✗ |
| Dick, 1975 [79] | South Africa | 67 | Before-after | Moraten | CF (>1:8) | ✓ | ✗ |
| Wallace, 1976 [104] | Nigeria | 1021 | Before-after | Schwarz | HAI (NR) | ✓ | ✗ |
| WHO, 1977 [71] | Kenya | 1087 | RCT | Schwarz | HAI (1:3) | ✓ | ✗ |
| Guyer, 1977 [80] | Cameroon | 45 | Before-after | Schwarz | HAI (1:10) | ✓ | ✗ |
| Stewien, 1978 [94] | Brazil | 43 | Before-after | Schwarz | HAI (1:2) | ✓ | ✗ |
| Wilkins, 1979 [105] | USA | 851 | Before-after | Moraten | HAI (1:8) | ✓ | ✗ |
| Mittal, 1979 [95] | India | 34 | Before-after | Schwarz | CF (1:8) | ✓ | ✗ |
| Ogunmekan, 1981 [106] | Nigeria | 224 | Before-after | Schwarz | HAI (NR) | ✓ | ✗ |
| Ogunmekan, 1981b [107] | Nigeria | 98 | Before-after | Moraten | HAI (NR) | ✓ | ✗ |
| Shaoyuan, 1982 [96] | China | 1152 | Before-after | Jing55 | HAI (1:2) | ✓ | ✗ |
| De Haas, 1983 [108] | Tanzania | 681 | Before-after | Schwarz, Moraten | HAI (1:3) | ✓ | ✗ |
| Dequadros, 1983 [97] | Ecuador, Chile, Brazil | 2553 | Before-after | Moraten | HAI (1:10) | ✓ | ✗ |
| Lee, 1983 [128] | Taiwan | 124 | Before-after | Moraten | HAI (1:10) | ✓ | ✗ |
| Sehgal, 1983 [81] | India | 251 | Before-after | Schwarz | HAI (NR) | ✓ | ✗ |
| Climie, 1984 [109] | Papua New Guinea | 313 | Before-after | Schwarz | HAI (1:10) | ✓ | ✗ |
| Job, 1984 [110] | India | 278 | Before-after | Moraten | HAI (1:4) | ✓ | ✗ |
| Chen, 1985 [111] | Malaysia | 1495 | Before-after | Schwarz | HAI (1:10) | ✓ | ✗ |
| Ekunwe, 1985 [75] | Nigeria | 168 | Before-after | Moraten | HAI (1:10) | ✓ | ✗ |
| Halsey, 1985 [82] | Haiti | 595 | Before-after | Moraten | HAI (1:10) | ✓ | ✗ |
| Maluf, 1985 [84] | Brazil | 223 | Before-after | NR | HAI (1:10) | ✓ | ✗ |
| Saha, 1985 [83] | India | 769 | Before-after | Schwarz | HAI (1:16) | ✓ | ✗ |
| Soerensen, 1985 [92] | Brazil | 1268 | NRCT | Schwarz | HAI (1:8) | ✓ | ✓ |
| Diaz-Ortega, 1986 [123] | Mexico | 237 | Before-after | Schwarz | HAI (1:10) | ✓ | ✗ |
| McGraw, 1986 [85] | USA | 181 | NRCT | Moraten | HAI (1:10) | ✓ | ✓ |
| Swami, 1987 [86] | India | 69 | Before-after | Schwarz | HAI (1:16) | ✓ | ✗ |
| Gendrel, 1988 [87] | Gabon | 117 | Before-after | Schwarz | HAI (1:10) | ✓ | ✗ |
| Lhuillier, 1989 [112] | Ivory Coast | 410 | NRCT | Schwarz | HAI (1:10) | ✓ | ✗ |
| Tidjani, 1989 [113] | Togo | 1162 | NRCT | AIK-C | HAI (1:10) | ✓ | ✗ |
| Deivanayagam, 1990 [76] | India | 205 | Before-after | Schwarz | HAI (1:4) | ✓ | ✗ |
| Huang, 1990 [114] | Taiwan | 160 | NRCT | Schwarz | ELISA (10mIU/ml) | ✓ | ✗ |
| Kakakios, 1990 [98] | Australia | 425 | Before-after | Schwarz, Moraten | HAI (1:10) | ✓ | ✗ |
| Makino, 1990 [115] | Japan | 1369 | Before-after | AIK-C | HAI (NR) | ✓ | ✗ |
| Markowitz, 1990 [124] | Mexico | 1969 | NRCT | Schwarz, E-Z | PRN (40mIU/ml) | ✓ | ✗ |
| Jain, 1990 [88] | India | 179 | Before-after | NR | HAI (1:8) | ✓ | ✗ |
| Job, 1991 [89] | Haiti | 2097 | NRCT | Schwarz, E-Z | PRN (200mIU/ml) | ✓ | ✗ |
| Kiepiela, 1991 [116] | South Africa | 124 | NRCT | Schwarz, E-Z | ELISA (200mIU/ml) | ✓ | ✗ |
| Rogers, 1991 [99] | Papua New Guinea | 70 | NRCT | E-Z | ELISA (1:100) | ✓ | ✗ |
| Soula, 1991 [117] | Mali | 453 | NRCT | Schwarz | HAI (1:10) | ✓ | ✗ |
| Abanamy, 1992 [100] | Saudi Arabia | 105 | NRCT | Schwarz | IF (1:8) | ✓ | ✗ |
| Berry, 1992 [78] | Peru | 1033 | NRCT | E-Z | PRN (200mIU/ml) | ✓ | ✗ |
| Kaan, 1992 [77] | Kenya | 303 | Before-after | Schwarz | HAI (NR) | ✓ | ✗ |
| Giammanco, 1993 [118] | Italy | 99 | NRCT | E-Z | PRN (1:8) | ✓ | ✗ |
| Bolotovski, 1994 [125] | Uzbekistan | 3677 | NRCT | Schwarz, E-Z, AIK-C, Leningrad-16 | HAI (1:2) | ✓ | ✗ |
| Johnson, 1994 [119] | USA | 35 | NRCT | Moraten | MN (1:10) | ✓ | ✗ |
| Sakatoku, 1994 [129] | Ghana | 234 | Before-after | Schwarz | HAI (1:8) | ✓ | ✗ |
| Singh, 1994 [120] | India | 164 | NRCT | Schwarz | HAI (1:4) | ✓ | ✗ |
| Ndumbe, 1995 [126] | Cameroon | 258 | Before-after | Schwarz, Connaught | ELISA (200mIU/ml) | ✓ | ✗ |
| Adu, 1996 [90] | Nigeria | 1177 | NRCT | Schwarz, Biken-CAM, E-Z | HAI (1:10) | ✓ | ✗ |
| Hussey, 1996 [127] | South Africa | 88 | NRCT | Schwarz | PRN (200mIU/ml) | ✓ | ✗ |
| Markowitz, 1996 [72] | USA | 1008 | NRCT | Moraten | PRN (1:120) | ✓ | ✗ |
| Gans, 1998 [101] | USA | 87 | NRCT | Moraten | PRN (1:120) | ✓ | ✗ |
| Kumar, 1998 [121] | USA | 169 | NRCT | Moraten | MN (1:10) | ✓ | ✗ |
| Johnson, 2000 [122] | USA | 94 | NRCT | Moraten | MN (1:10) | ✓ | ✗ |
| Klinge, 2000 [7] | Germany | 129 | NRCT | Moraten | ELISA (200mIU/ml) | ✓ | ✗ |
| Gans, 2001 [6] | USA | 248 | NRCT | Moraten | PRN (120mIU/ml) | ✓ | ✓ |
| Garly, 2001 [73] | Guinea-Bissau | 1748 | RCT | Schwarz, E-Z | HAI (1:8) | ✓ | ✗ |
| Youwang, 2001 [132] | China | 503 | NRCT | Hu191 | ELISA (1:200) | ✓ | ✓ |
| Zanetta, 2001 [130] | Brazil | 552 | Before-after | Biken-CAM | ELISA (10 AU) | ✓ | ✗ |
| Gans, 2004 [93] | USA | 138 | NRCT | Moraten | PRN (1:4) | ✓ | ✓ |
| Redd, 2004 [8] | USA | 1026 | RCT | Moraten | ELISA (NR) | ✓ | ✗ |
| Borras, 2012 [91] | Spain | 61 | Before-after | Schwarz | ELISA (150mIU/ml) | ✓ | ✗ |
| Fowlkes, 2011 [68] | Malawi | 2200 | RCT | E-Z | ELISA (NR) | ✓ | ✗ |
| Vesikari, 2012 [69] | Finland, France, Germany | 1620 | RCT | Moraten | ELISA (255mIU/ml) | ✓ | ✓ |
| He, 2014 [70] | China | 280 | RCT | Hu191 | ELISA (200mIU/ml) | ✓ | ✓ |
| Martins, 2014 [74] | Guinea-Bissau | 909 | RCT | E-Z | HAI (125mIU/ml) | ✓ | ✓ |

Abbreviations: AU=arbitrary units; CF=complement fixation test; ELISA= enzyme immunoassays test; E-Z=Edmonston-Zagreb; HAI=hemagglutination inhibition test; IF=indirect fluorescent kit; IU=international units; LFA=live further attenuated; MN=micro neutralization assay; NA=not applicable, not possible to calculate; NR=not reported; NRCT=non-randomized controlled trial; PRN= plaque reduction neutralization test; RCT=randomized controlled trial; VE= vaccine efficacy; ✓=yes; ✗=non;

^1^Vaccination status ascertained by written records

^2^Measles cases confirmed by laboratory or clinical definition plus epidemiologically linked to another case; “Partially” means that only a proportion of cases were laboratory confirmed or had a clinical definition with an epidemiologic link.

^3^Overall vaccine efficacy calculated for one and two doses (according to the study)

^4^Total number of vaccinated participants

^5^Laboratory assay used and threshold for seropositivity
