# Supplementary material for: Effect of age at vaccination on the measles vaccine effectiveness and immunogenicity: systematic review and meta-analysis
Source: BMC Infect Dis. 2020 Mar 29;20:251. doi: 10.1186/s12879-020-4870-x (PMC7104533; doi:10.1186/s12879-020-4870-x)
Supplement: Supplementary file 3 — Additional file 3. Figure – Risk of bias of the observational studies. This figure represents the evaluation of the risk of bias (global and for each item) of the observational studies included in the analysis of measles protection. [file 12879_2020_4870_MOESM3_ESM.pdf]

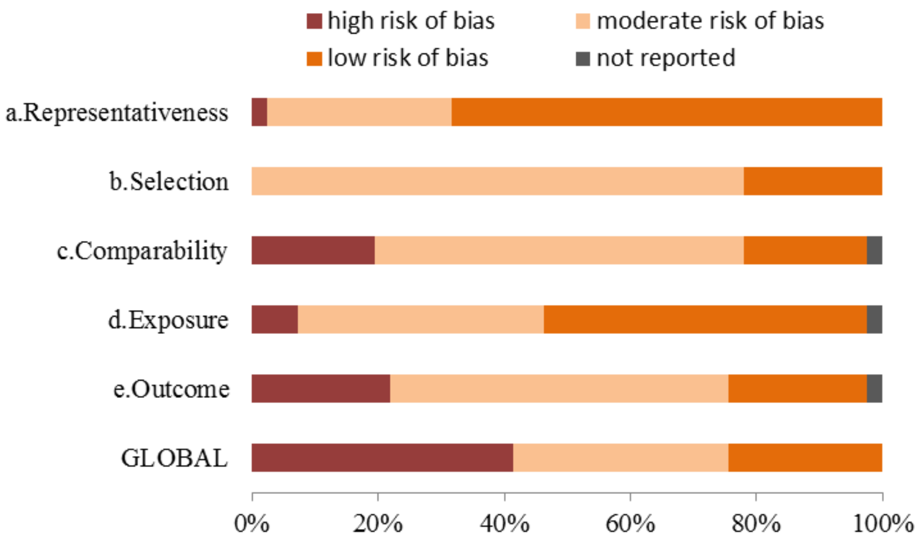

Supplementary Figure 1: Risk of bias of the observational studies included in the effectiveness analysis (n=41)

[a] Representativeness based on the description of the source population, the representativeness of the eligible population, the inclusion criteria and the percentage of participation.
[b] Selection of the compared groups from the same source and the response rate.
[c] Comparability of administered vaccine, time since vaccination, risk of exposure to measles, malnutrition and exposure to control measures between groups.
[d] Exposure assessment based on the method to ascertain vaccination status for all groups and the demonstration of no history of measles among no-cases.
[e] Outcome assessment based on the case definition and the completeness of case reporting.
