# Supplementary material for: Effect of age at vaccination on the measles vaccine effectiveness and immunogenicity: systematic review and meta-analysis
Source: BMC Infect Dis. 2020 Mar 29;20:251. doi: 10.1186/s12879-020-4870-x (PMC7104533; doi:10.1186/s12879-020-4870-x)
Supplement: Supplementary file 4 — Additional file 4. Figure – Risk of bias of the experimental studies. This figure represents the evaluation of the risk of bias (global and for each item) of the experimental studies (RCT and non-RCT) included in the immunogenicity analysis. [file 12879_2020_4870_MOESM4_ESM.pdf]

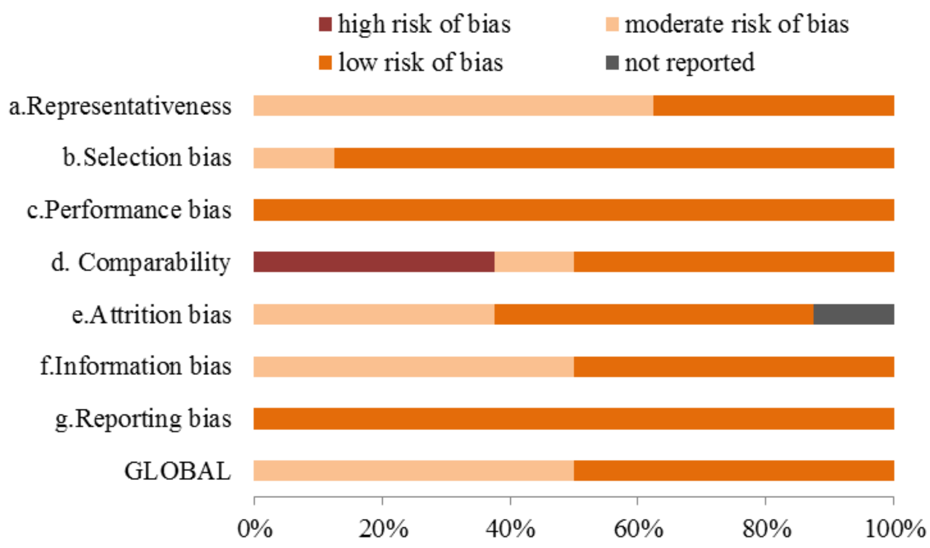

A. Randomized Controlled Trials (n=8)

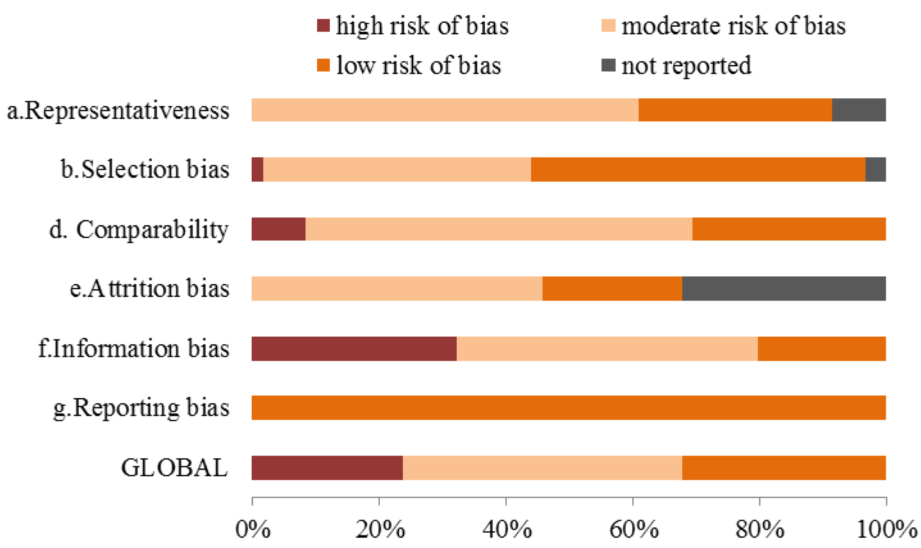

B. Non-randomized Controlled Trials / Before-after studies (n=59)

Supplementary Figure 2: Risk of bias of the experimental studies included in the immunogenicity analysis

[a] Representativeness based on the description of the source population, the representativeness of the eligible population, the inclusion criteria and the percentage of participation.
[b] Selection of the compared groups from the same source and the response rate.
[c] Blinding of study participants and personnel or no impact on the results
[d] Comparability of administered vaccine, time since vaccination, risk of exposure to measles, malnutrition and no control for maternal antibodies.
[e] Completeness of outcome data.
[f] Information bias assessment based on same vaccine strain and potency administered to all participants and laboratory method to detect antibodies.
[g] Possibility of selective outcome reporting
