# Supplementary material for: Effect of age at vaccination on the measles vaccine effectiveness and immunogenicity: systematic review and meta-analysis
Source: BMC Infect Dis. 2020 Mar 29;20:251. doi: 10.1186/s12879-020-4870-x (PMC7104533; doi:10.1186/s12879-020-4870-x)
Supplement: Supplementary file 5 — Additional file 5. Table – Sensitivity analysis with studies at low risk of bias. This table shows the effect estimate for each outcome including only the studies evaluated as having a low risk of bias. [file 12879_2020_4870_MOESM5_ESM.docx]

**Supplementary Table 2. Sensitivity analysis of all outcomes including only studies at low risk of bias**

| **Comparison**:  Age at MCV1 in months | **Outcome** | **Sub-analysis** (seroconversion definition) | **Number of studies** | | **I^2^** | | **Effect estimate** [95% CI] | |
| --- | --- | --- | --- | --- | --- | --- | --- | --- |
|  |  |  | All | Low Risk of Bias (ref) | All | Low Risk of Bias | All | Low Risk of Bias |
| <9 vs 12-14 | Measles RR |  | 3 | 1 [65] | 0% | NA | 3.56 [1.28, 9.88] | 0.63 [0.03, 14.59] |
| 9-11 vs 12-14 | Measles RR |  | 4 | 1 [65] | 39% | NA | 1.04 [0.45, 2.44] | 3.05 [0.38, 24.22] |
| <12 vs 12-14 | Measles RR |  | 13 | 1 [65] | 41% | NA | 1.62 [1.08, 2.43] | 2.42 [0.30,19.25] |
| ≥15 vs 12-14 | Measles RR |  | 16 | 1 [60] | 29% | NA | 0.48 [0.36, 0.63] | 0.60 [0.27, 1.32] |
| <12 vs 12-14 | Measles OR |  | 9 | 5 [4, 46, 57, 58, 63] | 0% | 0% | 1.34 [1.19, 1.51] | 1.03 [0.75, 1.41] |
| ≥15 vs 12-14 | Measles OR |  | 9 | 5 [4, 46, 57, 58, 63] | 74% | 48% | 0.25 [0.17, 0.37] | 0.31 [0.19, 0.50] |
| <9 vs 12 | Seroconversion PR | 4-fold | 4 | 4 [93, 66, 97, 101] | 65% | 65% | 0.74 [0.63, 0.87] | 0.74 [0.63, 0.87] |
| <9 vs 12 | Seroconversion PR | +/- PRE | 5 | 1 [6] | 54% | NA | 0.65 [0.55, 0.76] | 0.80 [0.64, 0.99] |
| <9 vs 12 | Seroconversion PR | 4-fold & +/- PRE | 1 | 1 [70] | NA | NA | 0.99 [0.97, 1.02] | 0.99 [0.97, 1.02] |
| 9-11 vs 12 | Seroconversion PR | 4-fold | 4 | 4 [93, 66, 97, 101] | 0% | 0% | 0.94 [0.91, 0.97] | 0.94 [0.91, 0.97] |
| 9-11 vs 12 | Seroconversion PR | +/- PRE | 10 | 3 [6, 69, 72] | 74% | 90% | 0.93 [0.89, 0.98] | 0.96 [0.87, 1.07] |
| 9-11 vs 12 | Seroconversion PR | 4-fold & +/- PRE | 1 | 1 [8] | NA | NA | 0.92 [0.87, 0.96] | 0.92 [0.87, 0.96] |
| ≥15 vs 12 | Seroconversion PR | 4-fold | 1 | 0 | NA | NA | 1.01 [0.96, 1.06] | NA |
| ≥15 vs 12 | Seroconversion PR | +/- PRE | 5 | 1 [122] | 53% | NA | 1.04 [0.98, 1.10] | 1.04 [0.96, 1.14] |
| ≥15 vs 12 | Seroconversion PR | 4-fold & +/- PRE | 1 | 1 [8] | NA | NA | 1.03 [1.00, 1.06] | 1.03 [1.00, 1.06] |

Abbreviations: CI= Confidence interval; 4-fold= fourfold increase in paired samples pre and post-vaccination; +/- PRE= seropositivity among seronegative pre-vaccination; MCV1= First dose of measles-containing vaccine; NA=Not applicable; OR= Odds Ratio; PR= Prevalence Ratio; RR= Risk Ratio
