# Supplementary material for: Effect of age at vaccination on the measles vaccine effectiveness and immunogenicity: systematic review and meta-analysis
Source: BMC Infect Dis. 2020 Mar 29;20:251. doi: 10.1186/s12879-020-4870-x (PMC7104533; doi:10.1186/s12879-020-4870-x)
Supplement: Supplementary file 6 — Additional file 6. Table – Seroconversion after one dose of MCV by age at vaccination. This table describes the seroconversion risk by age at vaccination of all studies included in the one-dose immunogenicity analysis. [file 12879_2020_4870_MOESM6_ESM.pdf]

Supplementary Table 3. Seroconversion after one dose of measles vaccine by age at first vaccination

<table>
<tr><th rowspan="2">Author, year
(strain) (ref)</th><th rowspan="2">N[1]</th><th rowspan="2">Serocon
-version
dfn[2]</th><th colspan="9">Seroconversion risk by age at MCV1[3] [95%CI]</th></tr>
<tr><th><6</th><th>6</th><th>7-8</th><th>9</th><th>10-11</th><th>12</th><th>13-14</th><th>15</th><th>>15</th></tr>
<tr><td>Stewien, 1978
(Schwarz) [108]</td><td>38</td><td>4-fold</td><td></td><td></td><td>0.42
[0.14-0.70]</td><td colspan="2">0.79
[0.61-0.97]</td><td>1.00
[-]</td><td></td><td></td><td></td></tr>
<tr><td>Mittal, 1979
(Schwarz) [109]</td><td>23</td><td>4-fold</td><td></td><td></td><td></td><td></td><td colspan="2">0.82
[0.59-1]</td><td colspan="3">0.92
[0.76-1]</td></tr>
<tr><td>Shaoyuan, 1982[4]
(Jing55) [110]</td><td>82</td><td>4-fold</td><td colspan="2">0.20
[0.06-0.34]</td><td>0.46
[0.26-0.66]</td><td>0.75
[0.54-0.96]</td><td colspan="2">0.92
[0.76-1]</td><td></td><td></td><td></td></tr>
<tr><td>Dequadros, 1983
(Moraten) [111]</td><td>1633</td><td>4-fold</td><td></td><td>0.59
[0.54-0.64]</td><td>0.76
[0.73-0.80]</td><td>0.87
[0.83-0.91]</td><td>0.94
[0.91-0.96]</td><td>0.97
[0.94-1]</td><td></td><td></td><td></td></tr>
<tr><td>Ekunwe, 1985
(Moraten) [52]</td><td>108</td><td>4-fold</td><td></td><td>0.74
[0.61-0.88]</td><td>0.79
[0.65-0.93]</td><td colspan="2">0.86
[0.71-1]</td><td colspan="4">0.87
[0.69-1]</td></tr>
<tr><td>Kakakios, 1990 [112]
(Schwarz/Moraten)</td><td>329</td><td>4-fold</td><td></td><td></td><td></td><td></td><td></td><td>0.97
[0.94-1]</td><td>0.94
[0.91-0.97]</td><td colspan="2">0.98
[0.95-1]</td></tr>
<tr><td>Rogers, 1991
(E-Z) [113]</td><td>39</td><td>4-fold</td><td>0.74
[0.58-0.91]</td><td>1.00
[-]</td><td colspan="7">1.00
[-]</td></tr>
<tr><td>Abanamy, 1992
(Schwarz) [114]</td><td>78</td><td>4-fold</td><td></td><td>0.56
[0.37-0.75]</td><td></td><td>0.70
[0.57-0.82]</td><td></td><td></td><td></td><td></td><td></td></tr>
<tr><td>Kaan, 1992
(Schwarz) [54]</td><td>170</td><td>4-fold</td><td colspan="3">0.58
[0.39-0.77]</td><td colspan="4">0.70
[0.62-0.79]</td><td colspan="2">0.79
[0.67-0.92]</td></tr>
<tr><td>Gans, 1998
(Moraten) [115]</td><td>65</td><td>4-fold</td><td></td><td>0.65
[0.46-0.85]</td><td></td><td>0.90
[0.77-1]</td><td></td><td>1.00
[-]</td><td></td><td></td><td></td></tr>
<tr><td>Gans, 2004
(Moraten) [73]</td><td>130</td><td>4-fold</td><td></td><td>0.86
[0.74-0.99]</td><td></td><td>0.95
[0.86-1]</td><td></td><td>0.98
[0.94-1]</td><td></td><td></td><td></td></tr>
<tr><td>Miller, 1967
(Schwarz) [116]</td><td>46</td><td>+/- PRE</td><td colspan="3">0.73
[0.46-0.99]</td><td colspan="2">1.00
[-]</td><td colspan="4">1.00
[-]</td></tr>
<tr><td>Ruben, 1973
(LFA) [117]</td><td>165</td><td>+/- PRE</td><td></td><td colspan="2">0.61
[0.47-0.76]</td><td colspan="6">0.81
[0.74-0.88]</td></tr>
<tr><td>Dick, 1975
(Moraten) [56]</td><td>64</td><td>+/- PRE</td><td></td><td>0.23
[0-0.46]</td><td>0.52
[0.32-0.72]</td><td colspan="2">0.81
[0.64-0.98]</td><td>0.80
[0.45-1]</td><td></td><td></td><td></td></tr>
</table>

Wallace, 1976<br>(Schwarz) [118]	136	+/- PRE			0.64<br>[0.47-0.80]			0.89<br>[0.83-0.95]			
Guyer, 1977<br>(Schwarz) [57]	21	+/- PRE		0.50<br>[0.22-0.78]				0.89<br>[0.68-1]			
Wilkins, 1979<br>(Moraten) [119]	851	+/- PRE		0.32<br>[0.16-0.49]	0.64<br>[0.50-0.78]	0.72<br>[0.60-0.84]	0.92<br>[0.86-0.98]	0.95<br>[0.93-0.97]	0.96<br>[0.91-1]	0.93<br>[0.79-1]	0.95<br>[0.82-1]
Ogunmekan, 1981<br>(Moraten) [120]	77	+/- PRE	0.06<br>[0-0.16]	0.25<br>[0.08-0.42]	0.40<br>[0.21-0.59]	0.60<br>[0.30-0.90]					
Ogunmekan, 1981b<br>(Schwarz) [121]	171	+/- PRE		0.56<br>[0.42-0.69]		0.55<br>[0.45-0.66]		0.40<br>[0.21-0.59]			
Shaoyuan, 1982[4]<br>(Jing55) [110]	1070	+/- PRE	0.64<br>[0.55-0.74]		0.84<br>[0.79-0.89]	0.94<br>[0.92-0.97]		0.97<br>[0.95-0.99]			
De Haas, 1983<br>(Sw/Moraten) [122]	56	+/- PRE			0.57<br>[0.31-0.83]	0.87<br>[0.73-1]			0.84<br>[0.68-1]		
Sehgal, 1983<br>(Schwarz) [123]	99	+/- PRE		0.80<br>[0.68-0.92]				0.83<br>[0.73-0.93]			
Climie, 1984[5]<br>(Schwarz) [124]	172	+/- PRE				1.00<br>[-]	0.96<br>[0.88-1]	1.00<br>[-]	1.00<br>[-]	0.97<br>[0.93-1]	
Job, 1984<br>(Moraten) [125]	181	+/- PRE		0.87<br>[0.80-0.94]		0.97<br>[0.93-1]		1.00<br>[-]			
Chen, 1985<br>(Schwarz) [126]	798	+/- PRE				0.95<br>[0.91-0.99]	0.95<br>[0.93-0.98]	0.99<br>[0.97-1]	0.98<br>[0.97-0.99]		
Halsey, 1985<br>(Moraten) [59]	333	+/- PRE		0.45<br>[0.31-0.59]	0.74<br>[0.65-0.83]	0.84<br>[0.75-0.94]	0.95<br>[0.90-0.99]	1.00<br>[-]			
Maluf, 1985<br>(NR) [61]	223	+/- PRE		0.50<br>[0.42-0.58]		0.52<br>[0.33-0.71]	0.77<br>[0.61-0.93]	0.70<br>[0.42-0.98]	0.80<br>[0.55-1]	0.81<br>[0.62-1]	
Soerensen, 1985<br>(Schwarz) [72]	1206	+/- PRE			0.53<br>[0.49-056]	0.75<br>[0.69-0.82]	0.79<br>[0.67-0.90]				
Lhuillier, 1989<br>(Schwarz) [127]	132	+/- PRE	0.96<br>[0.91-1]			0.98<br>[0.95-1]					
Tidjani, 1989<br>(AIK-C) [128]	130	+/- PRE	0.96<br>[0.93-1]			0.87<br>[0.73-1]					
Huang, 1990	160	+/- PRE						0.88		1.00	

(Schwarz) [129]								[0.82-0.95]		[-]	
Makino, 1990<br>(AIK-C) [130]	699	+/- PRE				1.00<br>[-]			0.996<br>[0.99-1]		
Jain, 1990<br>(NR) [65]	130	+/- PRE			0.54<br>[0.38-0.69]				0.64<br>[0.45-0.83]		0.64<br>[0.52-0.75]
Job, 1991<br>(E-Z) [66]	69	+/- PRE		0.76<br>[0.63-0.88]	0.96[6]<br>[0.88-1]						
Job, 1991<br>(Schwarz) [66]	86	+/- PRE		0.52<br>[0.38-0.67]	0.83[6]<br>[0.72-0.95]						
Kiepiela, 1991<br>(Schwarz; E-Z) [131]	55	+/- PRE	0.10<br>[0-0.29]	0.45<br>[0.16-0.75]	0.38<br>[0.12-0.65]	0.57<br>[0.36-0.78]					
Soula, 1991<br>(Schwarz) [132]	132	+/- PRE		0.83<br>[0.75-0.90]					0.98<br>[0.93-1]		
Giammanco, 1993<br>(E-Z) [133]	78	+/- PRE					0.98<br>[0.93-1]			0.94<br>[0.87-1]	
Johnson, 1994<br>(Moraten) [134]	33	+/- PRE		0.78<br>[0.59-0.97]						1<br>[-]	
Singh, 1994<br>(Schwarz) [135]	115	+/- PRE				0.80<br>[0.68-0.91]		0.98<br>[0.93-1]		0.95<br>[0.86-1]	
Adu, 1996 (Schwarz,<br>Biken-CAM,E-Z) [67]	143	+/- PRE		0.79<br>[0.63-0.95]			0.94<br>[0.90-0.98]				
Markowitz, 1996<br>(Moraten) [49]	303	+/- PRE				0.95<br>[0.91-0.99]		0.95<br>[0.92-0.98]			
Kumar, 1998<br>(Moraten) [136]	41	+/- PRE		0.81<br>[0.66-0.96]						1<br>[-]	
Johnson, 2000<br>(Moraten) [137]	93	+/- PRE						0.94<br>[0.82-0.99]		0.98<br>[0.88-1]	
Gans, 2001<br>(Moraten) [3]	187	+/- PRE		0.77<br>[0.61-0.93]		0.97<br>[0.92-1]		0.96<br>[0.91-1]			
Youwang, 2001<br>(Hu191) [138]	503	+/- PRE		0.81<br>[0.73-0.90]	0.91[7]<br>[0.84-0.98]			0.94[8]<br>[0.92-0.97]			
Borras, 2012<br>(Schwarz) [68]	31	+/- PRE				0.64<br>[0.44-0.84]		0.56<br>[0.23-0.88]			

Vesikari, 2012<br>(Moraten) [46]	1401	+/- PRE				0.73<br>[0.69-0.77]	0.88[9]<br>[0.85-0.91]	0.90<br>[0.87-0.93]			
Martins, 2014<br>(E-Z) [51]	489	+/- PRE	0.87<br>[0.82-0.92]			0.96<br>[0.93-0.98]					
WHO, 1977<br>(Schwarz) [48]	196	4 fold &<br>+/- PRE	0.59<br>[0.47-0.71]	0.57<br>[0.41-0.74]	0.93<br>[0.86-1]		1.00[9]<br>[-]				
Saha, 1985<br>(Schwarz) [60]	769	4 fold &<br>+/- PRE		0.33<br>[0.19-0.46]		0.53<br>[0.46-0.59]			0.42<br>[0.33-0.50]		0.41<br>[0.36-0.46]
Diaz-Ortega, 1986<br>(Schwarz) [139]	237	4 fold &<br>+/- PRE			0.55[6]<br>[0.33-0.77]	0.67<br>[0.54-0.80]	0.58<br>[0.46-0.70]		0.67<br>[0.54-0.81]	0.68<br>[0.55-0.80]	
Deivanayagam, 1990<br>(Schwarz) [53]	71	4 fold &<br>+/- PRE		0.48<br>[0.26-0.69]	0.50<br>[0.36-0.64]						
Markowitz, 1990<br>(Schwarz) [140]	274	4 fold &<br>+/- PRE	0.57<br>[0.49-0.65]		0.85<br>[0.79-0.91]						
Markowitz, 1990<br>(E-Z) [140]	322	4 fold &<br>+/- PRE	0.77<br>[0.70-0.84]		0.96<br>[0.94-0.99]						
Bolotovski, 1994<br>(AIK-C) [141]	281	4 fold &<br>+/- PRE		0.83<br>[0.77-0.88]		0.94<br>[0.89-0.97]					
Bolotovski, 1994<br>(E-Z) [141]	296	4 fold &<br>+/- PRE		0.67<br>[0.59-0.74]		0.91<br>[0.86-0.95]					
Bolotovski, 1994<br>(L16) [141]	288	4 fold &<br>+/- PRE		0.75<br>[0.68-0.81]		0.95<br>[0.91-0.98]					
Bolotovski, 1994<br>(Schwarz) [141]	301	4 fold &<br>+/- PRE		0.66<br>[0.58-0.73]		0.95<br>[0.91-0.98]					
Ndumbe, 1995<br>(Schwarz) [142]	45	4 fold &<br>+/- PRE	0.57<br>[0.39-0.75]	0.71<br>[0.49-0.92]							
Ndumbe, 1995<br>(Conraught) [142]	68	4 fold &<br>+/- PRE	0.59<br>[0.44-0.74]	0.82<br>[0.64-1]	0.83<br>[0.62-1]						
Klinge, 2000<br>(Moraten) [4]	118	4 fold &<br>+/- PRE				0.85<br>[0.74-0.95]		0.97<br>[0.90-1]		1<br>[-]	
Redd, 2004<br>(Moraten) [5]	990	4 fold &<br>+/- PRE				0.87<br>[0.84-0.91]		0.95<br>[0.93-0.97]		0.98<br>[0.97-1]	
He, 2014	280	4 fold &			0.99[6]			0.99			

(Hu 191) [47]		+/- PRE			[0.97-1]			[0.96-1]			
Gendrel, 1988<br>(Schwarz) [64]	37	Other<br>(1)	0.45[10]<br>[0.23-0.67]	0.41[11]<br>[0.18-0.65]							
Hussey, 1996<br>(Schwarz) [143]	45	Other<br>(2)		0.92<br>[0.81-1]		0.95<br>[0.86-1]					
Lee, 1983<br>(Moraten) [144]	113	+		0.82<br>[0.64-1]	0.93<br>[0.84-1]	1<br>[-]	1<br>[-]	1<br>[-]			
McGraw, 1986<br>(Moraten) [62]	111	+			0.88<br>[0.77-0.99]	0.90<br>[0.80-1]	0.88<br>[0.76-1]			0.95<br>[0.86-1.04]	
Swami, 1987<br>(Schwarz) [63]	60	+		0.43<br>[0.22-0.64]			0.73<br>[0.54-0.91]			0.64<br>[0.42-0.87]	
Berry, 1992 (EZ)<br>[55]	239	+	0.67<br>[0.58-0.76]			0.94<br>[0.90-0.98]					
Sakatoku, 1994<br>(Schwarz) [146]	159	+	0.46<br>[0.31-0.60]	0.87<br>[0.69-1]	0.93<br>[0.88-0.99]	0.92<br>[0.78-1]	0.91<br>[0.83-0.99]				
Garly, 2001<br>(EZ) [50]	292	+		0.95<br>[0.90-1]		0.99<br>[0.97-1]					
Garly, 2001<br>(Schwarz) [50]	416	+		0.78<br>[0.70-0.86]		0.97<br>[0.95-0.99]					
Zanetta, 2001<br>(Biken-CAM) [146]	290	+		0.31<br>[0.23-0.39]	0.44<br>[0.37-0.52]						
Fowlkes, 2011<br>(EZ) [45]	988	+		0.62<br>[0.58-0.66]		0.76<br>[0.72-0.80]					

Abbreviations: CI=Confidence interval; E-Z=Edmonston-Zagreb; NR=Not reported; MCV1= dose one of measles-containing vaccine

[1] Number of cases for the calculation of the age effect

[2] 4-fold= fourfold increase in paired samples pre and post-vaccination; +/-PRE= seropositivity among seronegative pre-vaccination; += seropositivity among all participants; other(1)= twofold increase; other(2)= fourfold rise two weeks after immunization or twofold rise three months after immunization or titer >200 mIU/ml;

[3] By age in months of administration of MCV1

[4] Data presented separately for seronegative and seropositive pre-vaccination

[5] Age categories: 8.5 to 9.4 months; 9.5 to 11.4 months; 11.5 to 12.4 months; 12.5 to 14.4 months; 14.5 to 23 months

[6]8 months
[7]7 months
[8]8 to 15 months
[9]11 months
[10]3 to 4 months
[11]5 to 7 months
