# Supplementary figures and images for: Effect of age at vaccination on the measles vaccine effectiveness and immunogenicity: systematic review and meta-analysis
Source: BMC Infect Dis. 2020 Mar 29;20:251. doi: 10.1186/s12879-020-4870-x (PMC7104533; doi:10.1186/s12879-020-4870-x)

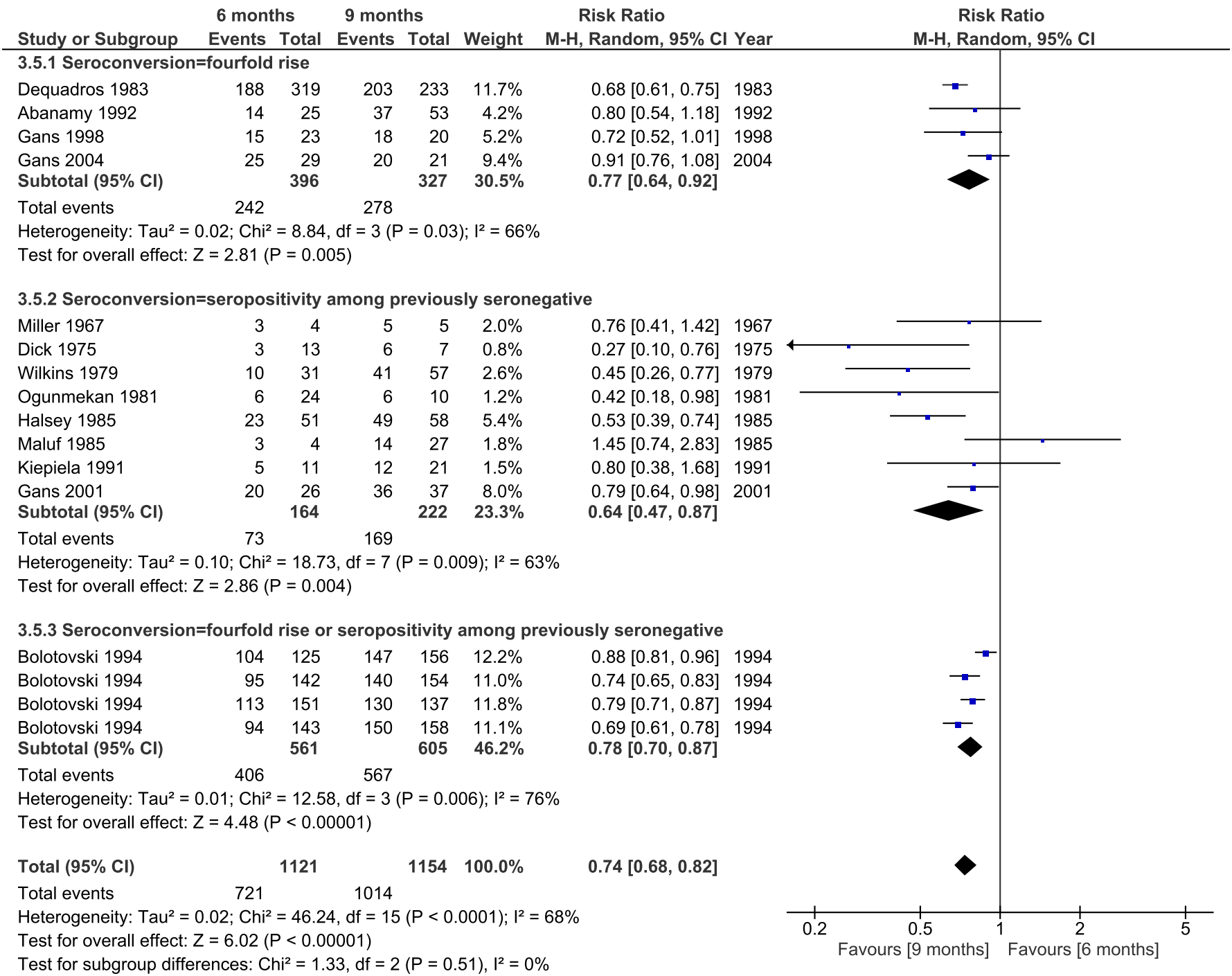

Supplement: Supplementary file 7 — Additional file 7. Figure – Seroconversion after one dose of MCV: 6 versus 9 months. This figure is a forest plot of the meta-analysis comparing seroconversion after one dose of MCV at 6 months versus 9 months of age. [file 12879_2020_4870_MOESM7_ESM.pdf]
