# Supplementary material for: Effect of age at vaccination on the measles vaccine effectiveness and immunogenicity: systematic review and meta-analysis
Source: BMC Infect Dis. 2020 Mar 29;20:251. doi: 10.1186/s12879-020-4870-x (PMC7104533; doi:10.1186/s12879-020-4870-x)
Supplement: Supplementary file 8 — Additional file 8. Figure – Funnel plots. This figure represents the funnel plots for each outcome. [file 12879_2020_4870_MOESM8_ESM.pdf]

A. Measles Risk Ratio

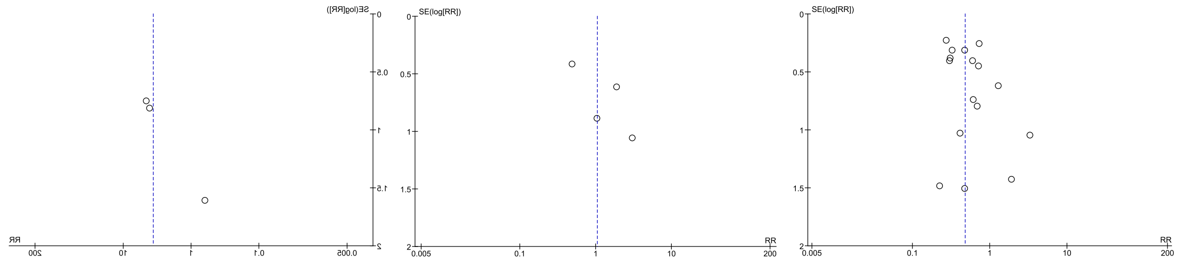

<9 versus 12 months | 9-11 versus 12 months | ≥15 versus 12 months

B. Measles Odds Ratio

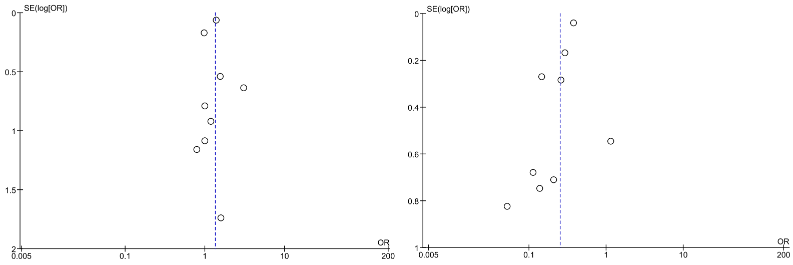

<12 versus 12 months | ≥15 versus 12 months

C. Seroconversion Prevalence Ratio

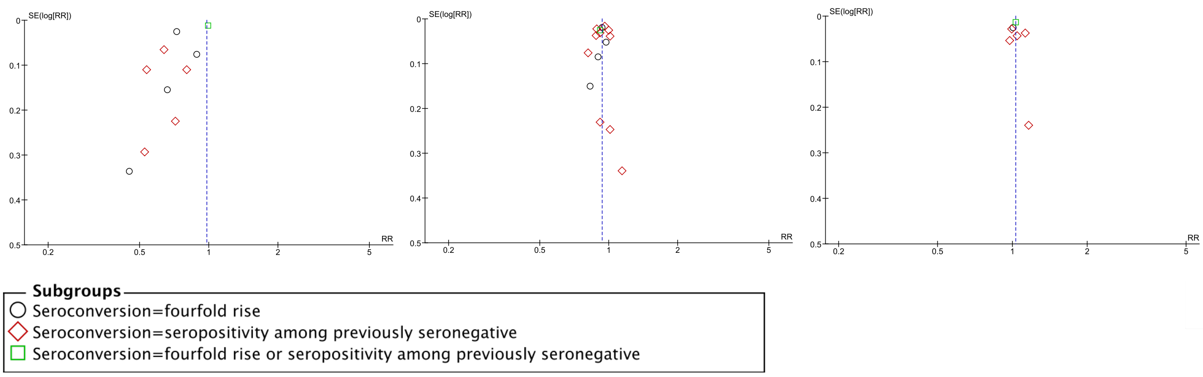

<9 versus 12 months | 9-11 versus 12 months | ≥15 versus 12 months

Supplementary Figure 5. Funnel plots for each outcome
